# Crosstalk between RNA secondary and three-dimensional structure prediction: a comprehensive study

**DOI:** 10.1080/15476286.2026.2655096

**Published:** 2026-04-01

**Authors:** Deyin Wang, Yangwei Jiang, Linli He, Linxi Zhang, Ruhong Zhou, Dong Zhang

**Affiliations:** a Institute of Quantitative Biology, School of Physics, and College of Life Sciences, Zhejiang University, Hangzhou, Zhejiang, China; b Department of Physics, Wenzhou University, Wenzhou, Zhejiang, China; c National Technology Innovation Center for Biopharmaceuticals, Suzhou, Jiangsu, China

**Keywords:** RNA 3D structure prediction, RNA 2D structure prediction, crosstalk, benchmarking, model improvement

## Abstract

In recent years, various computational methods have been developed to predict the three-dimensional (3D) structures of RNAs. Due to its hierarchical folding property, RNA secondary (2D) structure is often used as input for 3D structure prediction to improve accuracy and efficiency. However, the extent to which the accuracy of input 2D structure affects the performance of 3D structure prediction remains to be further investigated. Additionally, whether and how the input base-pairing interactions are modified during the 3D structure modelling process is another question worth exploring. To address these issues, here we comprehensively benchmark six representative 3D structure prediction models on extensive datasets, using 2D structures of varied accuracies as input. Our results indicate that there is a pervasive crosstalk between RNA 2D and 3D structure predictions, where the performance dependence of 3D structure prediction on the accuracy of input 2D structure is closely associated with the 3D model’s ability to modify the input base-pairing interactions during structure modelling. Furthermore, we also observed that RNA 3D structure prediction performance is more sensitive to the occurrence of false positive base pairs in the input 2D structure than to true positive base pairs, suggesting a worthy direction to further improve the model performance.

## Introduction

Ribonucleic acids (RNAs) are important functional macromolecules in living organisms. The particular three-dimensional (3D) structure adopted by most RNA molecules is typically crucial for their biological functions. However, determining the actual 3D structure of RNA often requires expensive and time-consuming experimental techniques. Therefore, computer programs for RNA 3D structure prediction have emerged in the past three decades [1,2]. Current RNA 3D structure prediction methods primarily fall into three categories: template-based methods, *de novo* methods, and the most recent deep learning-based methods. Template-based methods rely on known structures/fragments as templates to predict RNA 3D structures, including 3dRNA [3], ModeRNA [4], RNAComposer [5,6], Vfold3D [7], FARFAR2 [8], and more [9–14]. They search for templates with similar sequence or structural characteristics to model the 3D structure of the target molecule. On the other hand, *de novo* methods can predict the 3D structures of RNAs from scratch based on sequence information, with or without secondary (2D) structure as constraints, such as HiRE-RNA [15], IsRNA [16–18], iFoldRNA [19,20], SimRNA [21], and more [22–27]. They utilize physical and chemical principles to sample the conformational space and predict the most stable/probable conformation of the target molecule. Finally, inspired by the success of deep learning-based protein 3D structure prediction in the past few years [28–31], many deep learning-based methods have been developed to facilitate RNA 3D structure prediction, such as DRfold [32], RhoFold+ [33], RoseTTAFoldNA [34], trRosettaRNA [35], NuFold [36], GraphaRNA [37] and more [38,39]. Nevertheless, compared with protein structure prediction, these deep learning-based methods generally perform much worse for RNAs, indicating that RNA 3D structure prediction remains challenging [40].

In parallel, a collective and blind experiment called RNA-Puzzles was launched to assess the leading edge of RNA structure prediction technique [41–45]. Their results emphasized that computational methods for RNA structure prediction can already provide useful structural information for biological problems, but the poor prediction of non-Watson-Crick interactions suggests that the algorithms need further improvement. Meanwhile, in 2022, 12 experimental RNA targets were first introduced into CASP15 [46,47]. Two different assessments independently ranked four traditional methods (template-based or *de novo* methods) as top predictors, while deep learning-based approaches performed significantly worse than these top-ranked groups. Particularly, for synthetic RNA targets in CASP15 that lack homologous RNA sequences and structures akin to existing RNAs, their precise 3D structure modelling necessitates significant interventions from human experts [6,48–51]. Therefore, it is crucial to conduct more comprehensive benchmarking to identify the critical factors for predicting RNA 3D structures. Tentative benchmarking of RNA 3D structure prediction tools have been carried out recently [52–55], but these studies were limited by a narrow selection of RNA datasets or focusing exclusively on deep learning-based methods. We anticipate that insights gained from more comprehensive benchmark tests, combined with continued advances in computational biology, available datasets, and deep learning algorithms, may address the challenges of accurate RNA 3D structure prediction in the future.

In general, since RNA folding is hierarchical [56,57], its 2D structure is often used as a constraint in 3D structure modelling to improve prediction efficiency and accuracy. So far, numerous models have been proposed to obtain the RNA 2D structure from its sequence, such as RNAfold [58], RNAStructure [59], CONTRAfold [60], Mfold [61–63], HotKnots [64], MXfold2 [65], NUPACK [66], SPOT-RNA [67], PETfold [68], and more [69–76]. However, the 2D structure prediction accuracy of existing models is usually limited [77,78]. For example, on datasets containing diverse RNA sequences, the average F1-score of almost all tested models is less than 0.8 [65,67]. Thus, an extensive benchmark is required to examine how these predicted 2D structure inputs affect the prediction accuracy of current 3D structure methods. Furthermore, whether and how the input 2D structure is changed during the 3D structure modelling process is another question worth exploring.

Here, we comprehensively benchmark the performance of several representative RNA 3D structure prediction methods based on an extensive array of datasets, including RNAs considered in CASP15, CASP16, RNA-Puzzles, and a custom dataset composed of 30 curated RNAs. Our goals include: (1) comparing the prediction performances of typical RNA 3D methods, including template-based, *de novo*, and deep learning-based methods (see Table 1), using the native 2D structure derived from native 3D structures using DSSR (v2.4) [79] as input; (2) exploring the impact of 2D structure prediction accuracy on RNA 3D structure modelling, where 2D structures predicted by different models were used as inputs; (3) examining whether and how the raw 2D structure input is altered during the 3D structure modelling process. In addition to evaluating the prediction accuracy of different methods, we anticipate that the study of the crosstalk between RNA 2D and 3D structure predictions can reveal potential factors that affect the accuracy of RNA structure prediction, thereby paving the way for improving the accuracy and reliability of future computational models.

**Table 1.** Overview of RNA 3D structure prediction models tested in this study.

Model	Category	Access	Inputs
AlphaFold3 [31]	Deep Learning-based	Webserver	Seq
NuFold [36]	Deep Learning-based	Local	Seq + 2D
DRfold [32]	Deep Learning-based	Local	Seq
trRosettaRNA [35]	Deep Learning-based	Local	Seq + 2D
RNAComposer [5,6]	Template-based	Webserver	Seq + 2D
FARFAR2 [8]	Template-based	Local	Seq + 2D
IsRNA2 [18]	de novo	Local	Seq + 2D
SimRNA [21]	de novo	Local	Seq + 2D

## Methods and materials

The general process of predicting RNA 3D structure consists of two steps: (i) generating a 2D structure from the given RNA sequence using appropriate 2D structure prediction models, and (ii) predicting the 3D structure using the sequence and the generated 2D structure as input. Here the 2D structure contains only canonical base pairs (GC, AU, and GU). The RNA 2D and 3D structure prediction tools tested in this work are briefly introduced below.

### 2D structure prediction models

Six popular 2D structure prediction models were used to generate RNA 2D structures from sequence information in this work, including RNAfold [58], RNAStructure [59], CONTRAfold [60], Mfold [61–63], NUPACK [66], and MXfold2 [65]. These six models can be categorized into two groups: thermodynamics-based models and deep learning-based models. Their key features are displayed in Table 2, and detailed information is listed in Supplementary Information (SI) Table S1. In the following benchmarks, all the prediction packages were downloaded and run locally. For reference, the native 2D structures derived from experimental structures using DSSR (v2.4) [79] were also prepared as input and denoted as the *native* 2D structure in subsequent tables and figures. Although other annotation tools, such as RNAview [80], and MC-Annotate [81], can also extract 2D structures from experimental structures, for most RNAs, there is little to no difference in the annotations of Watson-Crick base pairs between different tools (see Fig S1). Therefore, given the widespread use of DSSR, this study selected and used DSSR throughout to ensure consistency of results. In addition, the 2D structures extracted from 3D structures predicted by AlphaFold3 [31] using DSSR (v2.4) were also considered as another baseline (denoted as AlphaFold3-2D).

**Table 2.** Summary of RNA 2D structure prediction models tested in this study.

Model	Category	Access
RNAfold [58]	Thermodynamics	Local
NUPACK [66]	Thermodynamics	Local
Mfold [61–63]	Thermodynamics	Local
RNAStructure [59]	Thermodynamics	Local
CONTRAfold [60]	Statistical models	Local
MXfold2 [65]	Deep Learning	Local

### 3D structure prediction methods

Considering the diversity of prediction categories (deep learning-based, template-based, and *de novo* methods), performance in CASP15, and the availability of support for diverse RNA 2D structure inputs, six representative RNA 3D structure prediction methods were benchmarked here, including trRosettaRNA [35], NuFold [36], RNAComposer [5,6], FARFAR2 [8], IsRNA2 [18], and SimRNA [21]. We select trRosettaRNA and NuFold as the representative deep learning-based methods due to their leading performance in a recent benchmark of RNA 3D structure prediction methods [54] and, more importantly, because they officially support customized 2D structure inputs. Additionally, AlphaFold3 [31] and DRfold [32] are also included as benchmark models when using native RNA 2D structures; however, they are not evaluated with predicted 2D structures because they do not officially support customized 2D structure inputs. A summary of these methods is shown in Table 1 and their brief features are listed in the supplementary information (SI) Text. Their corresponding run commands and the number of output predictions are listed in Table S2 for reference. For each prediction among the selected 3D models using different 2D structures as input, the top five predictions (when available) were collected by local runs (trRosettaRNA, DRfold, NuFold, FARFAR2, IsRNA2, and SimRNA) or by the webserver (AlphaFold3, RNAComposer). The best prediction among the collected candidates was used for subsequent studies.

### Test datasets

To comprehensively evaluate the performance of selected RNA 3D structure prediction methods, we constructed three test datasets, including ‘*Custom*’, ‘*RNA Puzzles*’, and ‘*CASP RNA*’. To construct the *Custom* dataset, we first collected curated RNA-only structures released between March 2021 to December 2024 from the database *RNAsolo* [82] (available at https://rnasolo.cs.put.poznan.pl/). The corresponding sequences were then clustered using Cd-hit-est [83] at an 80% sequence similarity threshold. Finally, after manual selection, a custom dataset containing 30 RNA molecules was prepared. These 30 RNAs have varied lengths and structural topologies; for more details, see Table S3 in the SI. The *RNA Puzzles* dataset contains 18 RNAs collected from the real challenging RNA-Puzzles [41–45], see Table S4 for more details. The *CASP RNA* dataset covers 14 RNA targets used in the CASP15 and CASP16 competitions; see details listed in Table S5. Moreover, we merged these three test datasets (*Custom*, *RNA Puzzles*, and *CASP RNA*) to form a *Combined* dataset (62 RNAs). For in-depth analysis, we also reclassified the *Combined* dataset into three categories according to the structural topology, namely stem-loop (21 RNAs), multi-way junction (16 RNAs), and pseudoknot (25 RNAs). As shown in Fig S2, based on the native 2D structure, a nearly linear relationship between the number of nucleotides ($N_{\mathrm{nt}}$) and the number of canonical base pairs ($N_{\mathrm{pair}}$) was observed in the *Combined* dataset, indicating that almost all tested RNAs formed substantial 2D structures. In terms of structural topology, the proportions of stem-loop, multi-way junction, and pseudoknot RNAs are 33.9%, 25.8%, and 40.3%, respectively, declaring the richness and balance of structural types in the test datasets.

### Evaluation metrics

Same as in previous studies [65,67], the F1-score was used to evaluate the accuracy of RNA 2D structure prediction, with values between $\left[0,\;1\right]$ and F1-score = 1 indicating that the predicted 2D structure perfectly matches the native one. To comprehensively assess the accuracy of RNA 3D structure predictions, we considered multiple metrics including Root-Mean-Square-Deviation (RMSD) [84], Interaction Network Fidelity of all base-base interactions (INF_ALL) [85], Local Distance Difference Test (lDDT) [86], and TM-score [87]. See SI Text for their detailed definitions and calculations. Briefly, the values of INF_ALL, TM-score, and lDDT range from 0 to 1, where value of one indicates an ideal prediction. To further investigate the impact of the input 2D structure (canonical base pairs) on the accuracy of 3D structure prediction, two additional metrics were introduced, namely the proportion of true positive ($\alpha_{\mathrm{TP}}$) and false positive ($\alpha_{\mathrm{FP}}$) predictions, defined as

(1) $$\alpha_{\mathrm{TP}}=\frac{N_{\mathrm{TP}}}{N_{\mathrm{bp},\;\mathrm{native}}}$$

(2) $$\alpha_{\mathrm{FP}}=\frac{N_{\mathrm{FP}}}{N_{\mathrm{bp},\;\mathrm{native}}}$$

where $N_{\mathrm{TP}}$ and $N_{\mathrm{FP}}$ represent the number of true positive and false positive predicted canonical base pairs, respectively. $N_{\mathrm{bp},\mathrm{native}}$ denotes the number of canonical base pairs contained in the native 2D structure. The proportion $\alpha_{\mathrm{TP}}$ takes value in the range $\left[0,\;1\right]$, while $\alpha_{\mathrm{FP}}$ may exceed 1. One can get $\alpha_{\mathrm{FN}}+\alpha_{\mathrm{TP}}=1$ using the similar definition for the proportion of false negative ($\alpha_{\mathrm{FN}}$) base pairs, thus we will not discuss metric $\alpha_{\mathrm{FN}}$ additionally.

## Results

### Benchmark results for RNA 3D structure prediction based on native 2D structure

**Figure 1.**
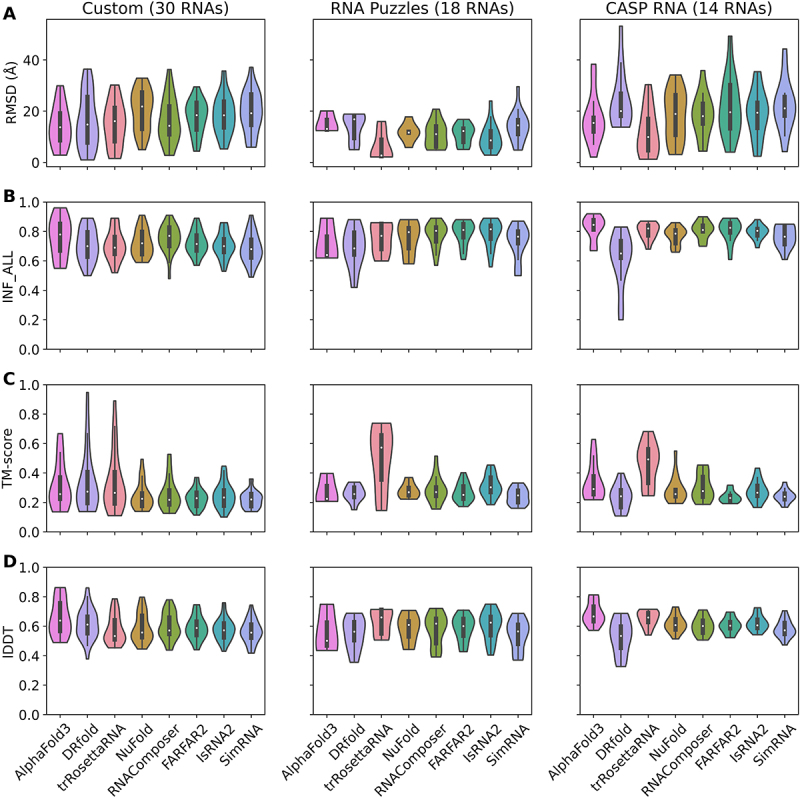
Benchmark results of RNA 3D structure prediction using the native 2D structure as input. violin plots of (A) RMSD, (B) INF_ALL, (C) TM-score, and (D) lDDT predicted by six selected 3D structure models. Three different test datasets were benchmarked: a custom dataset *custom* collected from PDB the real challenge *RNA puzzles* and *CASP RNA* the number of RNAs contained in each dataset is shown in parentheses.

To explore the prediction upper bounds of the six selected RNA 3D models (trRosettaRNA, NuFold, RNAComposer, FARFAR2, IsRNA2, and SimRNA), we first performed benchmark tests on three different datasets, including ‘*Custom*’, ‘*RNA Puzzles*’, and ‘*CASP RNA*’ (see Methods and Materials and Supplementary Information (SI) Tables S1-S3 for details), using the native 2D structure derived from experimental structures using DSSR (v2.4) [79] as input. To avoid potential data leakage, RNAs that appeared in the relevant training datasets of deep learning-based methods were excluded (see Table S6 for details) from the following results. As shown in Figure 1, for the *Custom* dataset, the template-based model RNAComposer provides the best predictions in terms of the RMSD metric, as it gives the lowest median RMSD = 14.3 Å (see Figure 1A), a relatively high median INF_ALL = 0.77 (see Figure 1B). However, FARFAR2 and trRosettaRNA provides the best predictions in terms of lDDT (median lDDT = 0.59, see Figure 1D) and TM-score (median TM-score = 0.27, see Figure 1B), respectively. These results re-emphasize the complexity of evaluating RNA 3D structure predictions and the necessity of using multiple metrics. For the *RNA Puzzles* dataset, trRosettaRNA provides the best predictions for almost all metrics among the six selected 3D models (median RMSD = 2.7 Å, median INF_ALL = 0.77, median TM-score = 0.57, and median lDDT = 0.66). For the *CASP RNA* dataset, trRosettaRNA also ranks top among the six selected models (median RMSD = 9.9 Å, median INF_ALL = 0.82, median TM-score = 0.49, and median lDDT = 0.66). Furthermore, we also considered the predictions from AlphaFold3 and DRfold as references. AlphaFold3 showed leading performance on metric INF_ALL among *Custom* and *CASP RNA* datasets (see Figure 1). For the *Custom* dataset, DRfold showed leading performance on metric TM-score (median TM-score = 0.27). NuFold did not show advantages over the traditional methods across the tested datasets. The full list of RMSD and INF_ALL for all tested methods is available in Tables S7-8. As AlphaFold3 webserver and DRfold do not officially support customized 2D structure inputs, we exclude these two models from further in-depth analysis. Overall, although the prediction performance of the 3D models for different RNA targets varied when using the native 2D structures as input, the prediction results of traditional methods (without human efforts) and recent deep learning-based approaches are comparable, which is consistent with the observations in the CASP15 competition [46].

### Benchmark results based on 2D structures predicted by different models

Now we consider a more general RNA 3D structure prediction scheme: using a pre-generated 2D structure as input to predict the possible 3D conformation of the query sequence. Six popular 2D structure prediction tools (see Table 2 for details) were tested to generate the input 2D structure. To facilitate subsequent analysis, we first investigated the 2D structure prediction accuracy of different 2D models. Since the RNA structure prediction accuracy (both 2D and 3D) usually depends on its structural topology, here we merged the above three test datasets into a combined dataset and then reclassified them into three categories based on their native structural information for in-depth analysis: stem-loops, multi-way junctions, and pseudoknots (see Figure S2). As listed in Table 3, all six tested 2D tools showed limited prediction accuracy, indicating that accurate RNA 2D structure prediction remains challenging. For instance, the best mean F1-scores are 0.640 (by Mfold), 0.795 (by MXfold2), and 0.705 (by MXfold2) for stem-loops, multi-way junctions, and pseudoknots, respectively. 2D structures derived using DSSR (v2.4) from AlphaFold3 (overlaps between *RNA Puzzles* and the training dataset not excluded) RNA 3D structure predictions exhibited significantly higher accuracy across all three structural categories, particularly for multi-way junctions and pseudoknots (with mean F1-score > 0.9). Regardless, due to their relatively high accuracy, AlphaFold3-derived 2D structures were also used as another baseline input (in addition to the native 2D structures) to extend our benchmark results in subsequent studies. As shown in Figure S3, the combination of different 2D and 3D structure prediction models leads to varied RNA 3D structure prediction performances. Systematic analysis of these combination results can provide useful guidance for constructing a well-tuned automated RNA 3D structure modelling pipeline.

**Table 3.** Summary of F1-scores for predictions from different 2D models. Mean values for RNAs with different topologies were displayed. The number of RNAs involved in each category is given in parentheses.

	Stem-loop (21)	Multi-way junction (16)	Pseudoknot (25)
RNAfold	0.609	0.723	0.637
NUPACK	0.516	0.546	0.597
Mfold	0.640	0.749	0.641
RNAStructure	0.630	0.757	0.646
CONTRAfold	0.574	0.724	0.696
MXfold2	0.633	0.795	0.705
AlphaFold3	0.791	0.917	0.940

**Figure 2.**
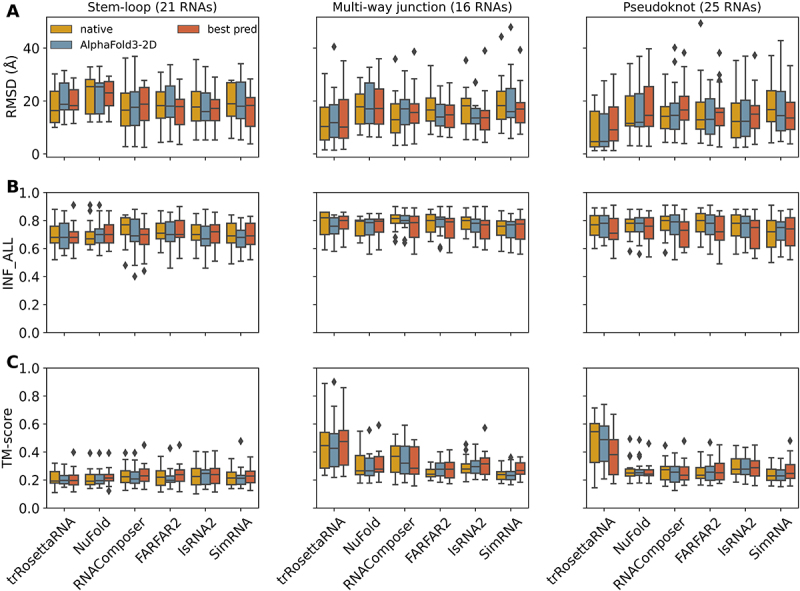
Summary of RNA 3D structure prediction performance using different 2D structures as input. Box plots of (A) RMSD, (B) INF_ALL, and (C) TM-score values for the predictions of six selected 3D models on different topological categories: stem-loop RNAs RNAs containing multi-way junctions and RNA pseudoknots predictions using native 2D structures as input are denoted as ‘native’, and ‘best pred’ represents the best prediction among all 2D methods for each case under each 3D structure prediction model. The whiskers of each box plot extend to the most extreme data points within 1.5 times the interquartile range (IQR) from the lower and upper quartiles, respectively. Observations beyond this range are plotted individually as outliers (grey diamonds).

As shown in Figure 2, when using predicted 2D structures as input, the selected 3D models performed similarly on stem-loops with median RMSD = 17.2–18.8 Å (see Figure 2A), median INF_ALL = 0.68–0.72 (see Figure 2B), and median TM-score = 0.2–0.24 (see Figure 2C), except for NuFold (with median RMSD = 23.1 Å, median INF_ALL = 0.7, and median TM-score = 0.21). While for multi-way junctions and pseudoknot RNAs, trRosettaRNA clearly outperformed the other models, with median RMSD = 10.1 and 9.1 Å (see Figure 2A), respectively. We also noticed that 3D structure predictions on stem-loops are slightly worse than those on multi-way junctions and pseudoknots, i.e. the median RMSD value for stem-loops is relatively large, even though the latter two categories are generally more structurally complex than the former. The relatively high proportion of unpaired nucleotides in the tested stem-loop RNAs (see Figure S2C) may be responsible for this phenomenon.

Notably, for almost all six selected 3D models, RNA 3D structure predictions using the predicted 2D structures as input generally performed worse than predictions based on the native 2D structures, i.e. the former usually have relatively higher median RMSDs, relatively lower median INF_ALL and TM-score values. Specifically, for RNAComposer, IsRNA2 and SimRNA, the difference in prediction performance between the two cases (using the native *vs*. predicted 2D structure as input) is significant on all three structural categories; while for FARFAR2 and SimRNA on stem-loop RNAs the difference is less pronounced (see Figure 2). However, for trRosettaRNA, NuFold, FARFAR2, IsRNA2, and SimRNA on multi-way junction RNAs and SimRNA on pseudoknot RNAs, as well as NuFold on stem-loop RNAs, we found that the best 3D prediction among predicted 2D structures as input outperformed those based on the native 2D structures. For the 2D inputs derived from AlphaFold3 predictions (denoted as AlphaFold3-2D in Figure 2), this phenomenon is more pronounced. In addition to the best performance over 3D predictions using predicted 2D structures by the six selected 2D tools as input, we also considered all 3D structure predictions based on predicted 2D structure or the AlphaFold3-derived 2D structure, and the results are shown in Figure S4. The median values of the results based on predicted 2D structure inputs are always worse than those based on native and AlphaFold3-derived 2D structure inputs. Overall, these results preliminarily demonstrated that different 3D models have varied dependencies on the accuracy of the input 2D structure.

### Dependence of 3D structure prediction performance on 2D structure accuracy

**Figure 3.**
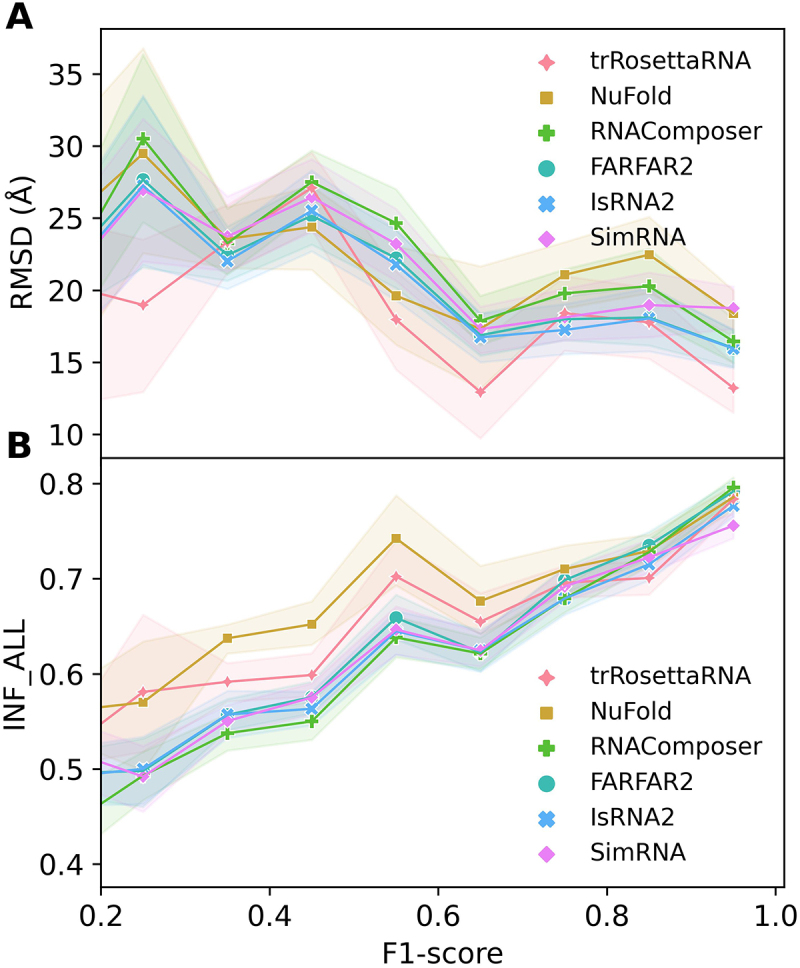
Accuracy of 3D structure predictions depends heavily on the accuracy of input 2D structures. (A) RMSD and (B) INF_ALL values of RNA 3D structures predicted by the six selected models as functions of the F1-score values of input 2D structures. The F1-score values were grouped by a bin size of 0.1. Symbols represent the mean metrics of a particular bin and shaded area indicates the 95% confidence interval. The analysis considered all 2D structures predicted by the selected six 2D tools, AlphaFold3-derived 2D structures, and the native 2D structures.

In general, the accuracy of 2D structures generated by currently available 2D tools is limited, and the accuracy may vary for different RNA sequences (see Table 3). Therefore, how these imperfect 2D structures (with F1-score < 1.0) as input affect the prediction performance of RNA 3D structure prediction methods is a question worthy of further exploration. To address this, we investigated the relationship between the RMSD/INF_ALL values of predicted 3D structures and the F1-score values of input 2D structures in the Combined dataset. As shown in Figure 3, for all six selected 3D methods, when the F1-score increases from 0.2 to 1.0, the overall trend of the RNA 3D structure prediction accuracy is on the rise, where RMSDs tend to decrease gradually (see Figure 3A) and INF_ALL values tend to increase gradually (see Figure 3B). Some local fluctuations on the profiles of RMSD/INF_ALL may be caused by the uneven distribution of different topological categories in particular bins of F1-score values (see Figure S5). In terms of individual test datasets, we observed similar variation trends for RMSD and INF_ALL in the *Custom*, *RNA Puzzles* datasets and INF_ALL in *CASP RNA* dataset (see Figure S6). These results indicated that, as expected, more accurate 2D structure as input can generally improve the performance of RNA 3D structure prediction. Furthermore, we found that NuFold significantly outperformed the other five 3D models in terms of INF_ALL in the interval of F1-score = 0.2–0.65. Meanwhile, trRosettaRNA also significantly outperformed the other four 3D models in terms of RMSD in the interval of F1-score = 0.55–0.65 and INF_ALL in the interval of F1-score = 0.2–0.65, suggesting the unique dependence on the accuracy of input 2D structure for deep learning-based models.

To further investigate to what extent the accuracy of the input 2D structure affects the performance of individual tested RNA 3D structure prediction models, we classified the predicted 2D structures into three categories according to their F1-score: low accuracy (F1−score<0.5), medium accuracy (0.5≤F1−score<0.9), and high accuracy (F1−score≥0.9). As shown in Figure S7, for 2D structures with low accuracy as input, the accuracy of 3D structure predictions for the majority of RNAs is generally poor. Among the six selected 3D models, the best median RMSD is 19.9 Å (obtained by IsRNA2), the highest median INF_ALL is 0.56 (obtained by trRosettaRNA), and the highest median TM-score is 0.17 (obtained by trRosettaRNA). In contrast, when using high-accuracy 2D structures as input, the accuracy of 3D structure predictions improves apparently, with the best median RMSD dropping to 11.8 Å (obtained by trRosettaRNA), the best INF_ALL increasing substantially to 0.81 (obtained by RNAComposer), and the best median TM-score also increasing to 0.44 (obtained by trRosettaRNA). Interestingly, when the accuracy of input 2D structure is moderate, the TM-score of trRosettaRNA is clearly better than that of the other four 3D models. Taken together, for the input 2D structure with medium accuracy, the 3D structure predicted by trRosettaRNA may have higher accuracy, which is in good agreement with results displayed in Figures 2 and 3.

**Figure 4.**
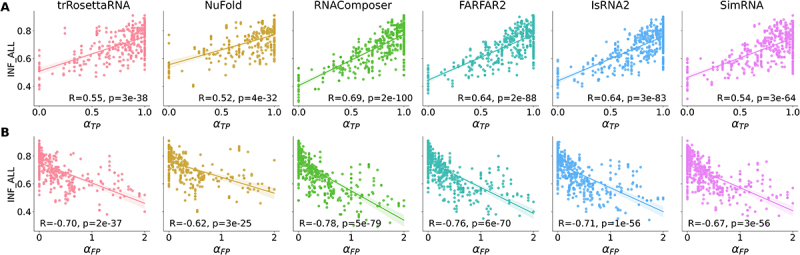
Different base pair compositions in the input 2D structure have distinct influences on the accuracy of 3D structure predictions. Scatter plots shown the relationship between the (A) proportion of true positive ($\alpha_{\mathrm{TP}}$) base pairs and (B) proportion of false positive ($\alpha_{\mathrm{FP}}$) base pairs in the input 2D structure and the INF_ALL values of 3D structures predicted by different 3D models. The line in each plane represents the linear fitting result, and the corresponding Pearson correlation coefficient (*R*) and *p*-value (*p*) are presented in the bottom. From left to right: trRosettarna, NuFold, RNAComposer, FARFAR2, IsRNA2, and SimRNA.

We considered two different types of base-pairing interactions in the predicted 2D structures: true positive base pairs that are correctly predicted and false positive base pairs that have no correspondence in the native structure. To deepen the understanding of the relationship between the accuracy of 3D structure prediction and the accuracy of input 2D structure, we then analysed the relationship between the prediction accuracy (characterized by RMSD and INF_ALL) and $\alpha_{\mathrm{TP}}$ (see Eq. 1) or $\alpha_{\mathrm{FP}}$ (see Eq. 2) for each tested 3D model. As shown in Figure 4, for all six selected 3D methods, the INF_ALL values are nearly positively correlated with $\alpha_{\mathrm{TP}}$ and negatively correlated with $\alpha_{\mathrm{FP}}$, which again declared that the accuracy of base pairs in the input 2D structure has a significant influence on the final accuracy of the predicted 3D structure. Expectedly, the positive correlation of INF_ALL with $\alpha_{\mathrm{TP}}$ and the negative correlation of RMSD with $\alpha_{\mathrm{TP}}$ (see Figure S8) indicated that the more correctly the base-pairing interactions in the input 2D structure were predicted, the higher the likelihood of achieving high-accuracy RNA 3D structure prediction. Conversely, the negative correlation of INF_ALL with $\alpha_{\mathrm{FP}}$ and the positive correlation of RMSD with $\alpha_{\mathrm{FP}}$ (see Figure S8) suggested that incorrectly predicted base-pairing interactions in the input 2D structure often lead to lower-accuracy 3D structure models. In general, those false positive base pairs introduce noise and mislead 3D structure modelling algorithms by suggesting incorrect folding patterns or interaction networks, especially for the template-based methods. Additionally, we observed that predictions by RNAComposer, FARFAR2, and IsRNA2 exhibited higher dependence of INF_ALL on $\alpha_{\mathrm{TP}}$ and $\alpha_{\mathrm{FP}}$ (Pearson correlation coefficient R≥0.64). Furthermore, although the number of false positive base pairs exceeds the number of native base pairs ($\alpha_{\mathrm{FP}}>1.0$) in some cases, the INF_ALL value did not display an abnormal drop (see Figure 4B), indicating the robustness of tested 3D models in structure prediction.

In addition to these global dependencies observed across the entire test dataset, investigating relationships for a particular RNA is also meaningful. Therefore, we selected R1108 from the *CASP RNA* dataset as an example to further examine the relationship between 3D structure prediction accuracy and the F1-score of input 2D structures across a wide range of predicted RNA 2D structures (see Figure S9). Using the same sequence but different 2D structures of varied accuracy as input, we observed similar dependencies consistent with those displayed in Figures 4 and S8: INF_ALL and TM-score are positively correlated with F1-score across all tested RNA 3D structure prediction models, while RMSD is negatively correlated with F1-score. This re-emphasizes that both the sequence and the input 2D structure simultaneously influence the performance of RNA 3D structure prediction, confirming that higher-accuracy 2D structures are helpful for accurate 3D structure modelling. Furthermore, these results partially alleviate concerns about data leakage between the training and test sets of the tested deep-learning base models (trRosettaRNA and NuFold), considering that only a limited number of 2D structures (if any) were used as input during model training, while the test results are based on a wide range of 2D structure inputs.

Moreover, RNA molecules involve many recurring motifs that contain specific non-Watson-Crick base pairing interaction networks, such as the GNRA tetraloop. This raises the question of whether RNA 3D structure prediction models can consistently preserve these particular structural elements (and adjacent Watson-Crick base pairs) regardless of the input 2D structure of varying accuracy. Therefore, for each tested RNA 3D structure prediction model, we calculated the proportion of Watson-Crick and non-Watson-Crick base pairs that were retained in more than 50% of the 3D structures predicted from different 2D structure inputs. As shown in Figure S10A, the median proportion of retained Watson-Crick pairs exceeds 0.5 for all tested RNA 3D structure prediction methods, indicating that most Watson-Crick base pairs are generally well preserved across tested models. In contrast, the proportions of retained non-Watson-Crick base pairs are substantially lower (Figure S10B). These results suggest that while the accuracy of input 2D structures is generally limited to canonical base pairs, it markedly affects the modelling of non-Watson-Crick interactions across different prediction methods. In other words, when using 2D structures with varying accuracies as input, although the adjacent canonical base-pairing patterns defining the boundaries of these motifs may be consistently preserved, the specific non-Watson-Crick base pairing interaction networks within them may be incorrectly modelled. We will conduct further in-depth research on this in the future.

### Modification of base-pairing interactions during 3D structure modelling

**Figure 5.**
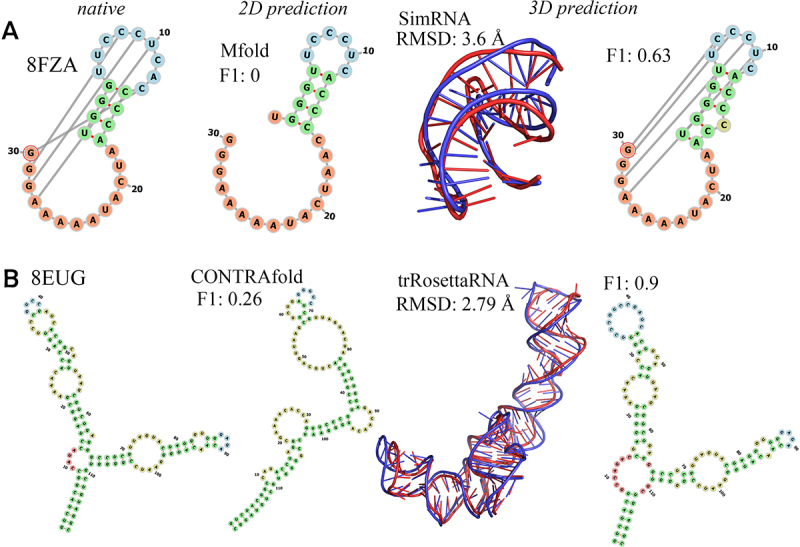
Illustrative examples of modifications of base-pairing interactions during 3D structure modelling when using an incorrected 2D structure as input. (A) The class I type iii preQ1 riboswitch from *E. coli* [88] (PDB id: 8FZA) and its 3D structure prediction by SimRNA. (B) The structure of Ytm1 associated nascent 60S ribosome state 3 [89] (PDB id: 8EUG) and its 3D structure prediction by trRosettarna. Columns from left to right: the native 2D structure extracted from experimental structure, predicted 2D structure by CONTRAfold as input, predicted 3D structure (in red) aligned with experimental structure (in blue), and modified 2D structure derived from the predicted 3D structure. Values of RMSD of 3D structure and F1-score of the corresponding 2D structure derived from corresponding 3D structure are also shown.

In Figure 4, we noticed that even if the true positive base pairs in the input 2D structure are zero ($\alpha_{\mathrm{TP}}=0$), most of the INF_ALL values of predictions from all six selected 3D models are non-zero, and some predictions even give INF_ALL > 0.5. This indicated that all these 3D models are able to (partially) modify the input base-pairing interactions during the 3D structure modelling process, e.g. identifying and forming correct base pairs. A similar phenomenon has also been reported in other study [35]. Two illustrative examples were presented in Figure 5 to further demonstrate this observation. The predicted 2D structures of these two RNAs as input deviate markedly from their corresponding native 2D structures (F1−score= 0 and 0.26, respectively). However, SimRNA (see Figure 5A) and trRosettaRNA (see Figure 5B) still generated 3D structure predictions for these two RNAs with F1−score = 0.63 and 0.9, respectively. These special cases showed that even if the input 2D structure is far from the native 2D structure, some key tertiary interactions can still be recovered during RNA 3D structure modelling. In addition, we also observed cases where base-pairing interactions deteriorated during 3D structure modelling; see SI Text and Figure S11 for more details.

**Figure 6.**
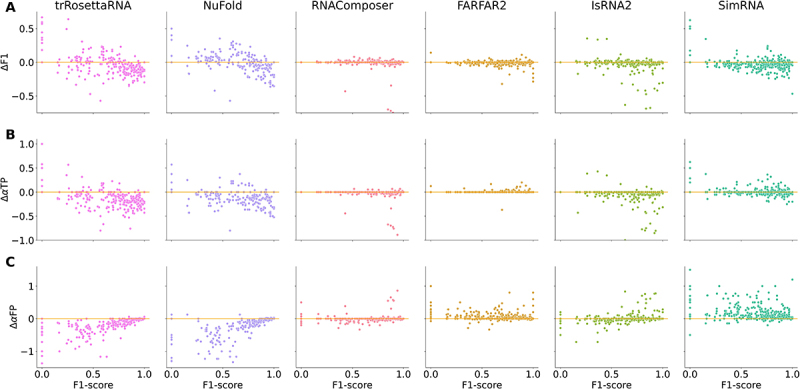
Different 3D models vary in their ability to modify input base-pairing interactions during structure prediction. scatter plots of the changes in (A) F1-score (ΔF1), (B) proportion of true positive ($\Delta \alpha_{\mathrm{TP}}$), and (C) proportion of false positive ($\Delta \alpha_{\mathrm{FP}}$) base pairs during 3D structure modelling *vs*. F1-score of the input 2D structure. Each point represents a 3D structure prediction using a 2D structure generated by one of the six selected 2D tools as input. Predictions with notably modified base-pairing interactions are marked with rectangular boxes. From left to right: trRosettarna, NuFold, RNAComposer, FARFAR2, IsRNA2, and SimRNA.

To gain a preliminary understanding of the ability of various 3D models to modify the input base-pairing interactions during RNA 3D structure modelling, we analysed the changes in the interaction network of predicted 3D structures using 2D structures of different accuracy as input. Figure 6 showed the changes in F1-score (ΔF1), proportion of true positive base pairs ($\Delta \alpha_{\mathrm{TP}}$), and false positive base pairs ($\Delta \alpha_{\mathrm{FP}}$) relative to the F1-score of input 2D structure during 3D structure modelling for all six selected 3D models. Overall, for trRosettaRNA and NuFold, many predictions improved their base-pairing interactions relative to the input 2D structure (ΔF1>0, see Figure 6A), especially for inputs with F1−score<0.8, which was achieved by the introduction of more true positive base pairs ($\Delta \alpha_{\mathrm{TP}}>0$, see Figure 6B) and/or the elimination of false positive base pairs ($\Delta \alpha_{\mathrm{FP}}<0$, see Figure 6C) during 3D structure modelling. We also observed similar results for some predictions of IsRNA2 and SimRNA when using low-accuracy 2D structures (F1−score<0.3, see Figure 6A) as input. Furthermore, when using high-accuracy 2D structures as input, we also found that some predictions from trRosettaRNA, NuFold, RNAComposer, IsRNA2, and SimRNA had obvious deteriorated base-pairing interactions (ΔF1<0), i.e. a decrease in true positive base pairs ($\Delta \alpha_{\mathrm{TP}}<0$) and an increase in false positive base pairs ($\Delta \alpha_{\mathrm{FP}}>0$); see Figure 6. These results indicated that modification of input base-pairing interactions during structure prediction is prevalent in the tested 3D models, and that this modification capability varies between different 3D models.

**Figure 7.**
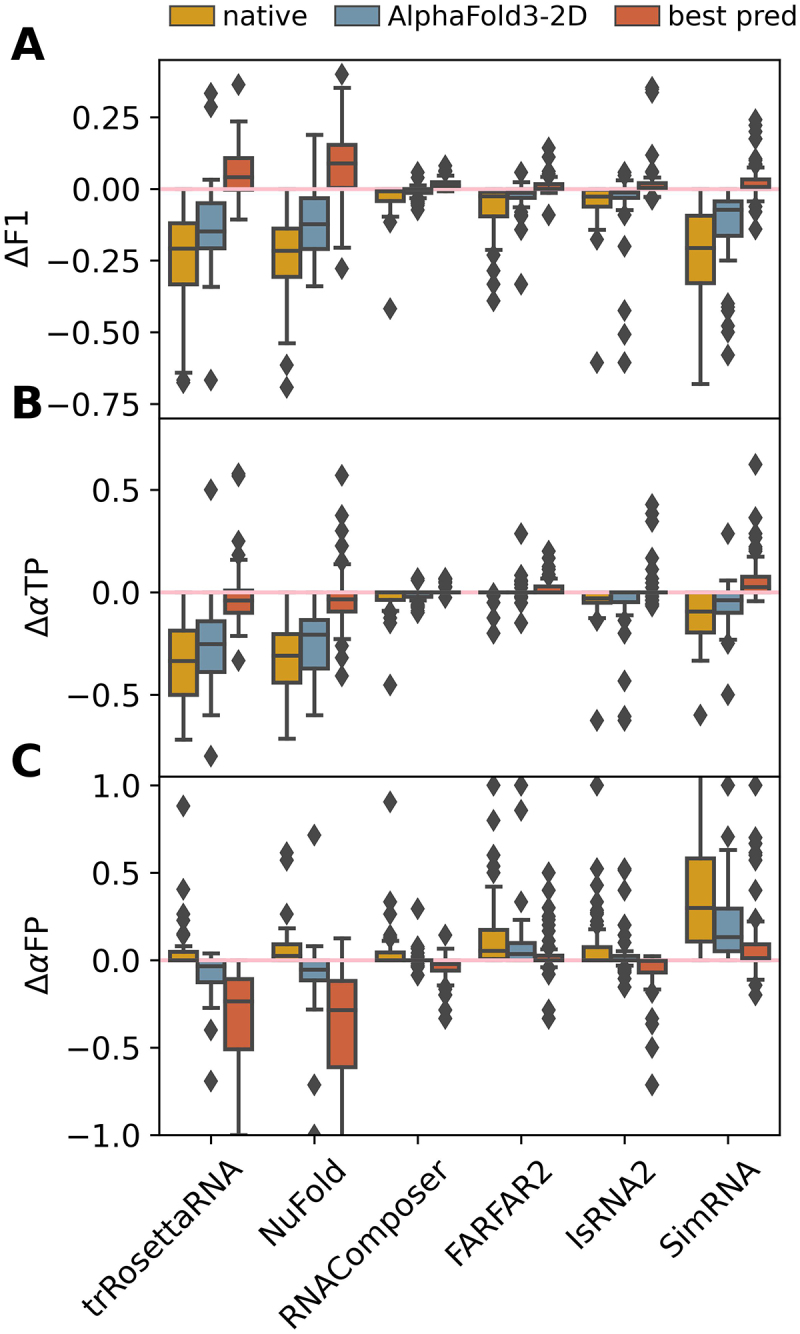
Accuracy of input 2D structures shown distinct effects on the ability of the six selected 3D models to modify base-pairing interactions. Box plots of (A) ΔF1, (B) $\Delta \alpha_{\mathrm{TP}}$, and (C) $\Delta \alpha_{\mathrm{FP}}$ when using different 2D structures as input for 3D structure predictions. Predictions using the native and AlphaFold3-derived 2D structures as inputs are denoted as ‘native’ and ‘AlphaFold3-2D’, respectively, while ‘best pred’ represents the best 3D prediction among all 2D inputs predicted by 2D methods for each RNA under each 3D structure prediction model. The whiskers of each box plot extend to the most extreme data points within 1.5 times the interquartile range (IQR) from the lower and upper quartiles, respectively. Observations beyond this range are plotted individually as outliers (grey diamonds).

Finally, we attempted to establish a link between the 3D structure prediction performance of each tested 3D model and its ability to modify input base-pairing interactions during the RNA 3D structure modelling process, namely cross-talk between RNA 2D and 3D structure predictions. To this end, we compared the changes in base- pairing interactions during 3D structure predictions when using different 2D structures as input, including the optimal 2D structure that produced the best prediction (in terms of RMSD) among the six selected 2D tools, the AlphaFold3-derived high-accuracy 2D structure, and the native 2D structure. As shown in Figure 7, for the template-based RNAComposer, the ability to modify the input base-pairing interactions was negligible in most predictions (median ΔF1 ∼ 0.0), regardless of whether the AlphaFold3-derived or native 2D structures were used as input. This point is consistent with the above observation that the performance of RNAComposer is heavily dependent on the accuracy of input 2D structure. For the deep learning-based methods trRosettaRNA and NuFold, we observed clearly distinct results when using predicted 2D structures as input. Specifically, when the optimal predicted 2D structure (usually with medium accuracy) was used as input, some predictions of trRosettaRNA and NuFold have improved accuracy of base-pairing interactions in many cases by eliminating some erroneous base pairs during structure modelling (median $\Delta \alpha_{\mathrm{FP}}=-0.24$ for trRosettaRNA and − 0.29 for NuFold, see Figure 7C). This signature distinguishes deep learning-based methods from the other four tested 3D models, which may explain the unique dependences of trRosettaRNA and NuFold on the accuracy of input 2D structure in the interval of F1-score = 0.2–0.65 (see Figure 3). This phenomenon could be the reason that, for multi-way junction and pseudoknot RNAs, some models perform better with predicted 2D structures as input than with native ones (see Figure 2). However, when using the AlphaFold3-derived or native 2D structures as input, 3D structure predictions by trRosettaRNA, NuFold and SimRNA worsened the input base-pairing interactions (median ΔF1<−0.1, see Figure 7A) in many cases through excluding some true positive base pairs (median $\Delta \alpha_{\mathrm{TP}}<-0.2$ for trRosettaRNA and NuFold and −0.04 for SimRNA, see Figure 7B) and/or introducing some false positive base pairs (median $\Delta \alpha_{\mathrm{FP}}>0.1$ for SimRNA, see Figure 7C). The improvement in base-pairing accuracy of deep learning-based models may stem from the training data distribution, since these models are typically optimized to tolerate or adjust imperfect secondary structures rather than strictly preserve near-native ones. In contrast, the degradation of base-pairing accuracy under high-quality 2D inputs likely arises from an inconsistency between the modelling priors of 3D predictors and the externally supplied secondary structure constraints, leading to over-correction and sampling-induced disruption of true base pairs.

Furthermore, we also compared the F1-scores between the predicted 3D structures and their corresponding 2D inputs and observed consistent results. Namely, all six selected 3D models could present predictions where the F1-score of generated 3D structure is greater than the corresponding 2D input (in a fraction of 0.25–0.54; see Fig S12). As shown in Fig S12, predictions of NuFold had the most cases (fraction = 0.54) where the F1-score of predicted 3D structure is greater than the 2D input. Meanwhile, predictions of RNAComposer had the most cases (fraction = 0.54) where the F1-score did not change, while the predictions of trRosettaRNA and NuFold had the least cases (fraction = 0.07 and 0.09, respectively). Overall, these results declared that the crosstalk between RNA 2D and 3D structure predictions is pervasive and that the dependence of 3D structure prediction performance on the accuracy of input 2D structure is closely associated to the model’s ability to modify the input base-pairing interactions.

## Discussions and conclusions

For large-sized RNAs with complex structural topologies and/or long unpaired loops, accurate 3D structure prediction remains challenging even with the native 2D structure as input. In general, large-sized RNAs often possess complicated interaction networks beyond the standard Watson-Crick base-pairing interactions, making it difficult to accurately describe these interactions *via* scoring/energy functions (known as the scoring problem). In addition, RNA molecules are highly flexible and can fold into a vast number of possible conformations, making it computationally expensive to find an appropriate final structure (known as the sampling problem). As a result, the folding free energy landscape of complex RNA structures is very rugged with multiple local minima, making it challenging for current computational algorithms to identify the global minimum corresponding to the native conformation. Therefore, although we have recently witnessed remarkable progress in deep learning-based structural modelling of biological macromolecules, even the state-of-the-art methods, including the recent AlphaFold3 [31], still cannot achieve a level of accuracy in RNA 3D structure modelling comparable to that of protein structure prediction [28,30].

Due to the complexity of RNA folding, many deep learning-based methods and majority of traditional approaches tend to use 2D structures as input to improve the accuracy of 3D structure prediction, although end-to-end methods that directly generate 3D structures from sequences also exist (e.g. AlphaFold3). High-confidence 2D structures can be generated using existing 2D tools, multiple sequence alignments, or RNA foundation models. Moreover, for RNAs that adopt alternative functional 3D structures (such as riboswitches), using different 2D structures along with the same sequence as input is an effective way to address the nature of 3D conformational diversity. Our study found that the crosstalk between the input 2D structure and the resulting 3D structure prediction is prevalent in all tested 3D models. That is, the performance of RNA 3D structure prediction is highly dependent on the accuracy of the input 2D structure, and the 3D models are able to modify the original base-pairing interactions contained in the input 2D structure during the 3D structure modelling process. For instance, 3D structure prediction can improve the interaction network through introducing additional true positive base pairs and eliminating some false positive base pairs in the input 2D structure; and *vice versa*. We also observed a close relationship between the accuracy dependence of 3D structure prediction on input 2D structure and the ability of the 3D models to modify the input base-pairing interactions. For example, the template-based RNAComposer has a weak ability to modify the input base-pairing interactions, so its 3D structure prediction performance is heavily dependent on the accuracy of input 2D structure; while the deep learning-based trRosettaRNA and NuFold have a more significant ability to modify the input base-pairing interactions, especially for input 2D structures of medium and high accuracy, thus its performance dependence on the accuracy of input 2D structure is distinct (see Figures 6 and 7 for details). Therefore, when predicted 2D structures with moderate accuracy are used as input, trRosettaRNA and NuFold can achieve higher accuracy for some targets, likely because they better capture base-pairing interactions during the structure modelling process.

Regardless, the high-accuracy 2D structure as input is one of the keys to improving the accuracy of RNA 3D structure prediction. Although there may be an upper limit, when the accuracy of input 2D structure exceeds this limit, it has no effect or even a detrimental impact on the 3D structure prediction. Our study also suggests that the performance of 3D structure prediction is more sensitive to the occurrence of false positive base pairs in input 2D structure than to the true positive base pairs, which was indicated by the larger magnitude of Pearson correlation coefficients for the former in each 3D model (see Figures 4 and S7). In addition, the modification of false positive base-pairing interactions is more likely to occur (see Figures 6 and 7) during the 3D structure modelling process. Therefore, a worthy study direction to further improve the performance of 3D structure prediction is to minimize the occurrence of incorrectly predicted base-pairing interactions (reducing false positives) without compromising the presence of correct interactions (maintaining or slightly increasing true positives). Finally, similar to the idea proposed by previous studies [90,91], we anticipate that an iterative procedure integrating 2D and 3D structure predictions may be one of the promising solutions to simultaneously achieve accurate predictions of RNA 2D and 3D structures in the future.

Due to the large number of runs generated by combining different 2D and 3D structure prediction tools, as well as limitations in computational resources, we limited the number of prediction tools evaluated. Meanwhile, given the rapid development of RNA 3D structure prediction models, some of our conclusions may not be applicable to the latest methods. By analysing the potential correlation between changes in F1-scores and improvements in 3D accuracy (in terms of RMSD/TM-score) during 3D structure modelling, crosstalk between 2D and 3D structure predictions can be further investigated. However, since the capability to modify input base-pairing interactions is an inherent characteristic of each 3D model, and the accuracy of 3D structure prediction is affected by various factors (including but not limiting to the changes in F1-score) during 3D modelling, future research needs to conduct a method that can cleverly distinguish the influence of F1-score changes from the contributions by others. Nevertheless, based on an extensive evaluation of various combinations of 2D and 3D tools and the assessment of thousands of prediction results, with a particular focus on the variations of base paring interactions between the modelling processes, we believe that our findings may still offer useful reference points for the future development of RNA structure prediction tools.

## Supplementary Material

RNA_3D_benchmark_SI_20260309.docx

## Data Availability

Details of the test datasets are included in Supporting Information Tables S1-S3. Prediction results are available at https://github.com/DongZhangRNA/2D-and-3D-benchmark for community use.
