# Supplementary material for: Crosstalk between RNA secondary and three-dimensional structure prediction: a comprehensive study
Source: RNA Biol. 2026 Apr 1;23(1):1–18. doi: 10.1080/15476286.2026.2655096 (PMC13078229; doi:10.1080/15476286.2026.2655096)
Supplement: RNA_3D_benchmark_SI_20260309.docx [file KRNB_A_2655096_SM2396.docx]

Supplementary Information

**Crosstalk between RNA secondary and three-dimensional structure prediction: a comprehensive study**

Deyin Wang^1,†^, Yangwei Jiang^1,2,†^, Linli He^2,*^, Linxi Zhang^1,*^, Ruhong Zhou^1^, and Dong Zhang^1,3*^

^1^ Institute of Quantitative Biology, School of Physics, and College of Life Sciences, Zhejiang University, Hangzhou, Zhejiang 310058, China

^2^ Department of Physics, Wenzhou University, Wenzhou, Zhejiang 325035, China

^3^National Technology Innovation Center for Biopharmaceuticals, Suzhou, Jiangsu 215009, China

^†^These authors contributed equally: Deyin Wang and Yangwei Jiang

* To whom correspondence should be addressed: linlihe@wzu.edu.cn (L.H.); [lxzhang@zju.edu.cn](mailto:lxzhang@zju.edu.cn) (L.Z.); [zhangd_iqb@zju.edu.cn](mailto:zhangd_iqb@zju.edu.cn) (D.Z.)

## S1. Supplementary Text

**Brief features of selected 3D models.**

**AlphaFold3** [1] is a deep learning-based model developed for predicting the joint structure of complexes including proteins, nucleic acids, small molecules, ions and modified residues. With a diffusion-based architecture, AlphaFold3 enables high-accuracy modeling of biomolecular structures through a unified deep-learning framework. Notably, it also attains remarkable accuracy in predicting 3D structures of single RNA molecules. The source code of AlphaFold3 is available at https://github.com/google-deepmind/alphafold3. The webserver of AlphaFold3 is available at https://alphafoldserver.com/.

**trRosettaRNA** [2] is an automated deep learning-based approach developed for RNA 3D structure prediction. The prediction process of trRosettaRNA involves two steps: first, predicting 1D and 2D geometries using a transformer network; then, folding the 3D structure via energy minimization. trRosettaRNA outperforms many deep learning-based and traditional methods in CASP15 [3]. The source code of trRosettaRNA is available at https://yanglab.qd.sdu.edu.cn/trRosettaRNA/download/.

**DRfold** [4] is a deep learning-based method that predicts RNA 3D structures by combining end-to-end model and geometry potentials. DRfold employs a coarse-grained model focusing on specific RNA atoms (phosphate P, ribose C4’, and glycosidic N atoms of the nucleobase) to enhance training efficiency. DRfold uses the RNA sequence and its 2D structure to predict frame vectors and geometric constraints, which are then aggregated into a composite potential for RNA 3D structure reconstruction simulations using Arena [5] and OpenMM [6]. The source code of DRfold is available at https://zhanggroup.org/DRfold/DRfold.zip.

**NuFold** [7] is an end-to-end deep learning-based method that predicts the fully atomistic 3D structure of RNA from its sequence. By integrating the metagenomic MSAs and RNA 2D structure, it achieves higher accuracy than classical energy minimization approaches. The source code of NuFold is available at https://github.com/kiharalab/NuFold.

**RNAComposer** [8,9] is a template-based model. Its workflow begins with fragmenting a user-defined RNA 2D structure into elements. These fragments serve as the input for an automated search for corresponding tertiary structure elements in the RNA FRABASE dictionary which are imported from the parent RNA FRABASE 2.0 database [10]. The 3D structures of elements are chosen based on 2D structure topology, sequence similarity, pyrimidine/purine compatibility, source structure resolution, and energy. These 3D structure elements are aligned using common canonical base pairs and merged to form an initial RNA 3D structure. This structure undergoes refinement through energy minimization in torsion angle space and Cartesian coordinates, resulting in a high-quality RNA 3D model. The RNAComposer webserver is available at https://rnacomposer.cs.put.poznan.pl/.

**FARFAR2** [11] is an upgraded version of FARFAR [12], offering enhanced prediction speed and accuracy. FARFAR initially constructs an RNA structure by assembling three-nucleotide fragments from pre-existing RNA structures that have a matching sequence to the target [12]. FARFAR2 introduces many improvements such as enhanced library of fragments derived from the 2018 crystallographic database of 657 RNA structures [13], score filters that identify and discard poorly assembled conformations early in the process during fragment assembly, Monte Carlo (MC) moves that preserve the Watson-Crick geometry, and updated all-atom scoring function in energy minimization stage. FARFAR2 offers both a program for local implementation and an online webserver on the ROSIE platform [14,15]. The source code of FARFAR2 is available at https://www.rosettacommons.org/software/license-and-download.

**IsRNA2** [16] is a *de novo* RNA 3D structure prediction method based on a coarse-grained representation and molecular dynamics (MD) simulations, evolving from its predecessors, IsRNA [17] and IsRNA1 [18]. IsRNA2 represents each nucleotide with five coarse-grained beads and utilizes the iterative-simulated reference state approach [17] to accurately parameterize the energy functions for subsequent MD simulations. Its improved energy functions offer a more precise characterization of canonical base-pairing and base-stacking interactions, leading to a more effective and accurate exploration of the conformational space of tertiary folding of medium to large RNA molecules. The standalone package of IsRNA2 is available upon request.

**SimRNA** [19] is a *de novo* RNA 3D structure modeling method that utilizes a coarse-grained representation of RNA molecules, simplifying each nucleotide to five beads while retaining essential RNA characteristics. Its energy function is based on statistical potential terms that are calculated from the observed frequencies of different nearby structural patterns (e.g., base-base contacts and short backbone fragments) using a collection of RNA 3D structures from protein data bank (PDB). SimRNA employs the MC technique to sample the conformational space and uses the statistical potential to estimate the energy, thereby identifying conformations that are indicative of biologically significant structures. The standalone package of SimRNA is available at https://genesilico.pl/software/stand-alone/simrna.

The commands used to execute these RNA 3D structure prediction tools and the number of outputs for each prediction task are listed in Table S5.

**Approximate computational resource consumption of locally deployed RNA 3D structure prediction models**

For traditional models such as IsRNA2, FARFAR2, and SimRNA, a single task typically requires several to tens of hours to complete, and running the full benchmark on four 14-core (28-thread) compute nodes (Intel(R) Xeon(R) Gold 6132 CPU @ 2.60GHz) requires several weeks. In contrast, for deep learning–based models such as DRfold, trRosettaRNA, and NuFold, a single task requires only a few to several tens of minutes, and the complete evaluation can be finished within several days using a workstation with 16-core (32-threads) AMD Ryzen Threadripper PRO 3955wx CPU, 32 GB of Memory, and an NVIDIA RTX 3090 GPU.

**The RNA 2D prediction tools used in this work.**

In addition to the models used in the main text, including RNAfold [20], RNAStructure [21], CONTRAfold [22], Mfold [23–25], NUPACK [26], MXfold2 [27], we further included SPOT-RNA [28], UFold [29], CentroidFold [30], PETfold [31], BPfold [32], LinearFold [33], and EternaFold [34] to perform secondary structure prediction for R1107 in the *CASP RNA* dataset. The resulting predicted 2D structures were subsequently used as inputs for RNA 3D structure prediction, and the corresponding results are shown in Fig. S1. The associated raw data are available at https://github.com/DongZhangRNA/2D-and-3D-benchmark/tree/main/R1108. Detailed information of these RNA 2D structure prediction tools is provided in Table S6.

**Definition of evaluation metrics.**

The F1-score is determined by:

|  | $F1\text{-}\mathrm{score}=\frac{2\times\frac{\text{TP}}{\text{TP+FN}}\times\frac{\text{TP}}{\text{TP+FP}}}{\frac{\text{TP}}{\text{TP+FN}}+\frac{\text{TP}}{\text{TP+FP}}}$ | (1) |
| --- | --- | --- |

where TP (true positive) is the number of correctly predicted base pairs, FP (false positive) is the number of predicted base pairs with no correspondence in the reference structure, and FN (false negative) is the number of base pairs in the reference model that are not present in the predicted structure. The F1-score has values between $[0, 1]$, where value of one means an ideal 2D structure prediction. The INF is determined by

|  | $\text{INF=}\sqrt{\left( \frac{\text{TP}}{\text{TP+FP}} \right)\text{×}\left( \frac{\text{TP}}{\text{TP+FN}} \right)}$ | (2) |
| --- | --- | --- |

The INF takes values in the range $[0, 1]$, where 1 indicates a perfect match between the interaction pattern of the predicted model and the reference structure. All interactions are considered here (i.e., INF_ALL) through RNA-tools [35], including canonical and noncanonical base pairing and base-stacking interactions. The value of INF_ALL was calculated directly using the default settings of RNA-tools without any modification. RNA-tools is publicly available at https://rna-tools.online/tools. TMscore is a metric for assessing the topological similarity of two structures, where smaller distance errors are weighted more strongly than larger distance errors, making the score more sensitive to the global fold similarity than to the local structural variations [36]. TMscore has values in $(0,1]$, where 1 indicates a perfect match between two structures. Instead of using the original TMscore calculation program RNA-align [37], US-align [38] was taken here as the tool for TMscore calculation. The lDDT evaluates the conservation of local interactions and it also has a value between 0 and 1. The lDDT value closer to 1 suggests higher model quality and more accurate local structure, while scores near 0 indicate significant deviations from the reference structure. OpenStructure [39,40] was used to find lDDT for predictions and no stereochemical violation penalty was applied for RNA here [3].

**Poor 3D structure prediction based on high-accuracy 2D structure as input.** Sometimes, though the accuracy of the input 2D structure is very high (F1-score > 0.9), the quality of the predicted 3D structure is poor (with RMSD > 8 Å and INF_ALL < 0.75); see an illustrative example in Fig. S8. This illustrative RNA molecule is characterized by a notably small number of base pairs and a long unpaired loop that is usually highly flexible. This flexible loop leads to conformational complexity during sampling, making it difficult to accurately predict its 3D structure even using the native 2D structure as input. During the 3D structure prediction process, each 3D model may create a particular number of base pairs in this long loop region, but they have no correspondence in the native 2D structure (false positive prediction). As a result, a low INF_ALL value was obtained for the predicted 3D structures. These results indicate that although these 3D models have the ability to form new base pairing interactions, the predicted 3D structure do not always accurately reflect the real spatial arrangement and interaction network of RNA folding, especially in the absence of a real folding environment (such as protein binding).

## S2. Supplementary Figures


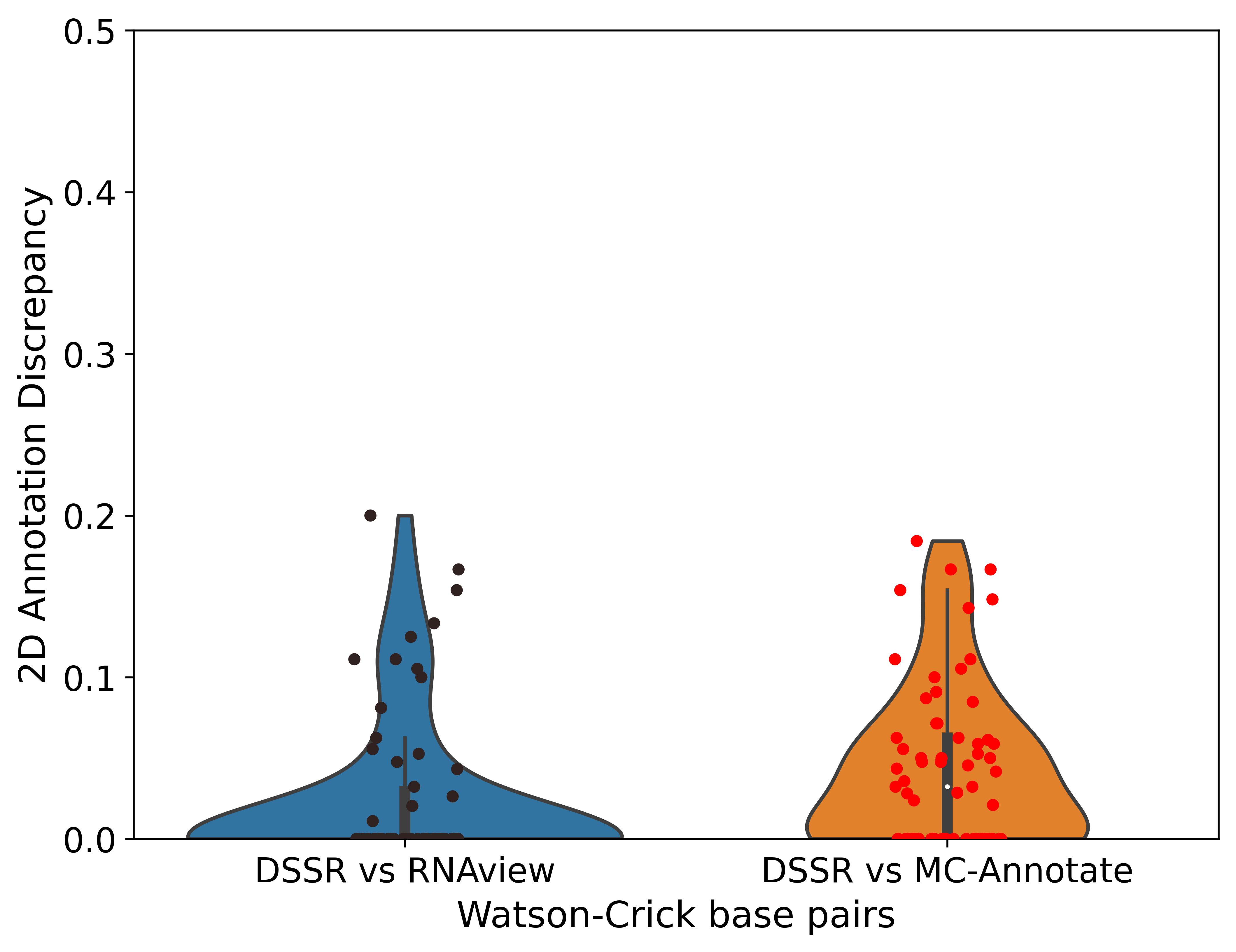


**Fig S1.** Violin plot showing the 2D annotation discrepancy of Watson-Crick (WC) base pairs between DSSR [41] and RNAview [42], and between DSSR and MC-Annotate [43]. Each point represents an RNA target in the *Combined* dataset. Discrepancy was defined as the proportion of non-overlapping base pairs relative to the union of annotated base pairs.


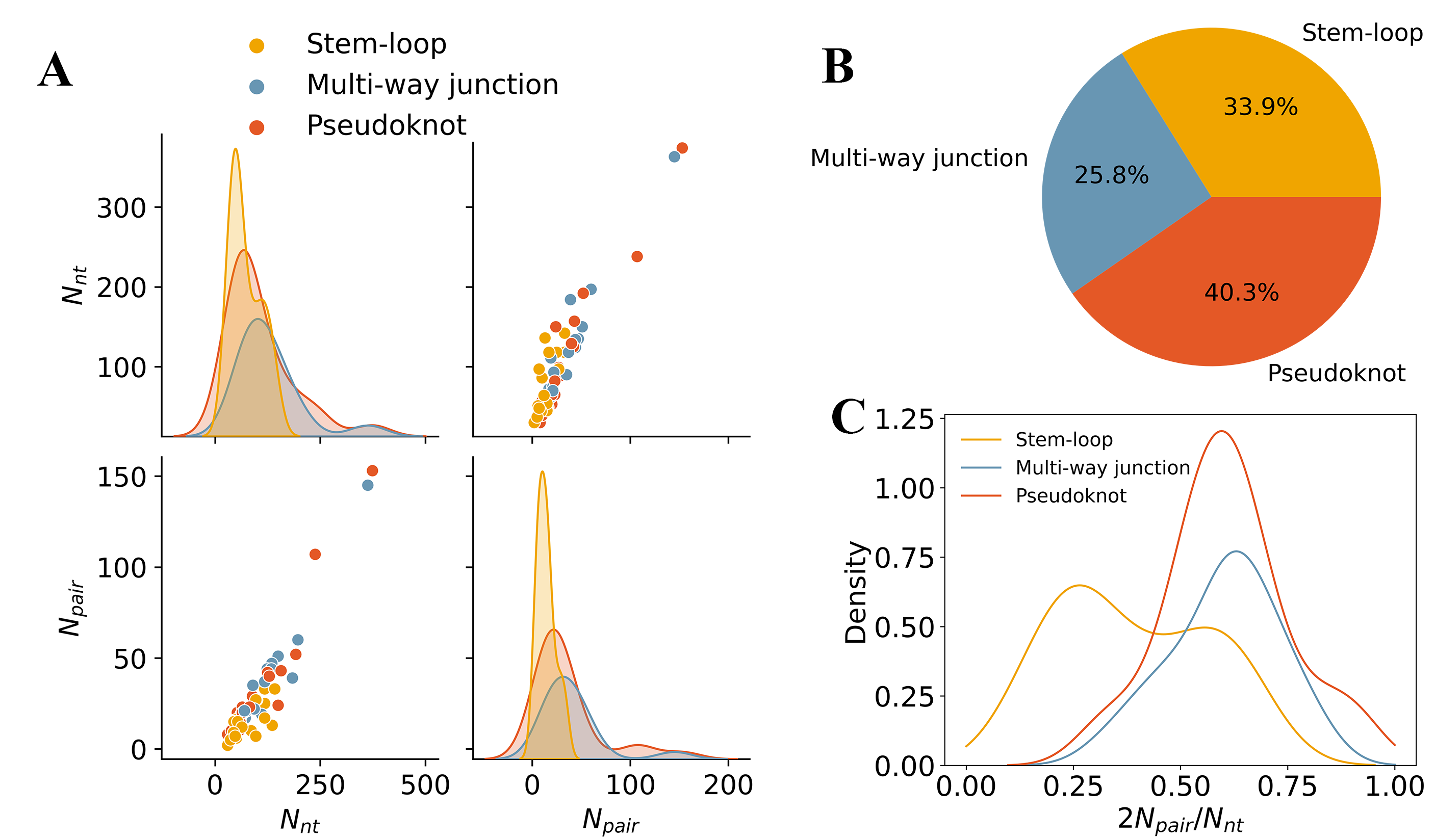


**Fig S2.** (A) Pair plots of the number of the nucleotides ($N_{nt}$) and the number of the canonical base pairs ($N_{pair}$) in the Combined dataset. (B) Pie chart for RNA structure topologies in the Combined dataset: stem-loop, multi-way junction, and pseudoknot (containing tertiary interactions). (C) Distributions of the proportions of paired nucleotides (${{2N}_{pair}}/{N_{nt}}$) in different structure topologies.


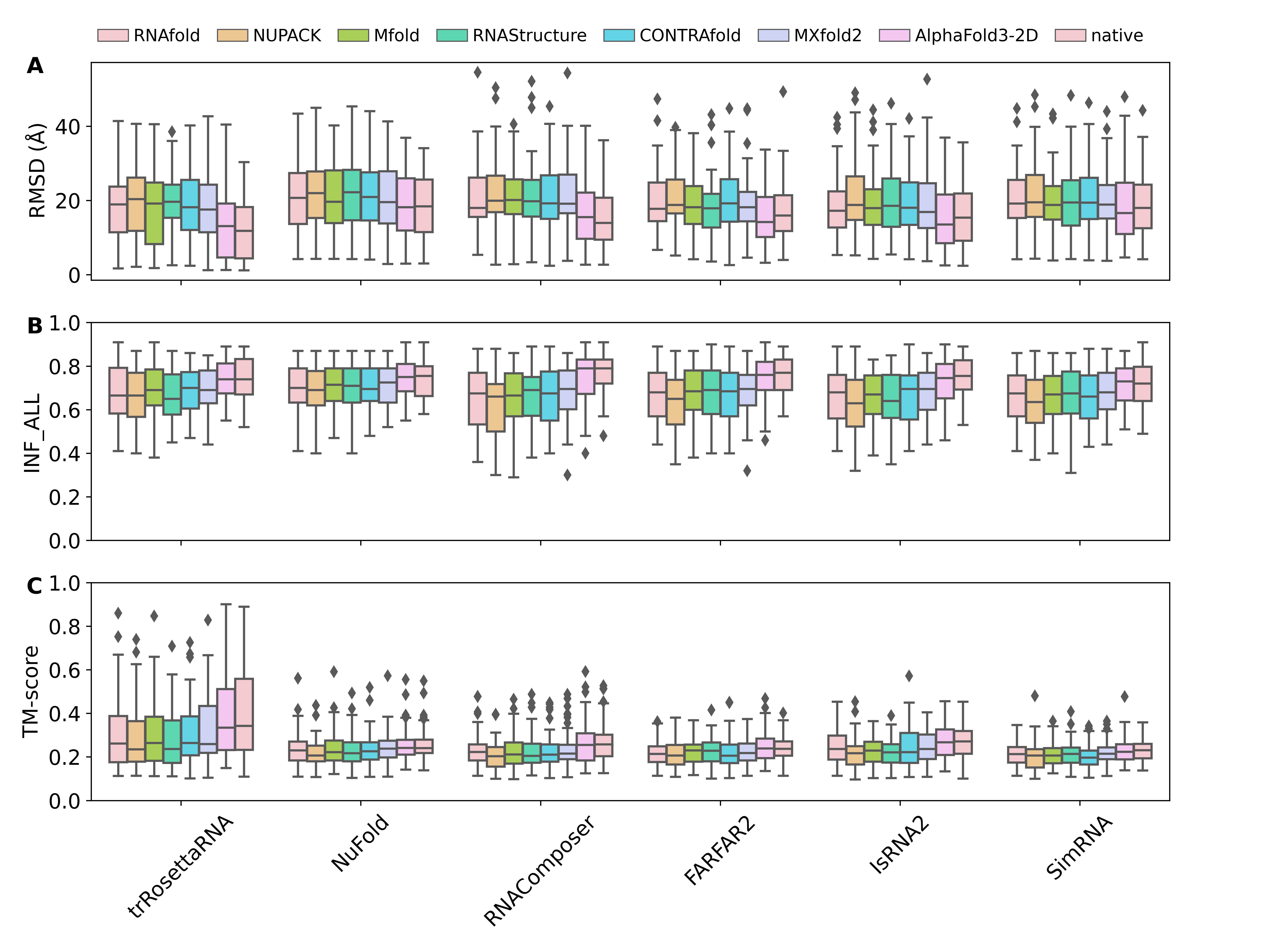


**Fig S3.** Box plots of (A) RMSD, (B) $INF\_ALL$, and (C) TMscore for different combinations of 2D and 3D structure predictions models in the Combined dataset. Individual 2D structure predicted by six popular 2D tools, the 2D structure derived from 3D structure predicted by AlphaFold3, or the native 2D structure was used as input for five selected 3D models to predict RNA 3D structure. From left to right: trRosettaRNA, RNAComposer, FARFAR2, IsRNA2, and SimRNA. The whiskers of each box plot extend to the most extreme data points within 1.5 times the interquartile range (IQR) from the lower and upper quartiles, respectively. Observations beyond this range are plotted individually as outliers (grey diamonds).


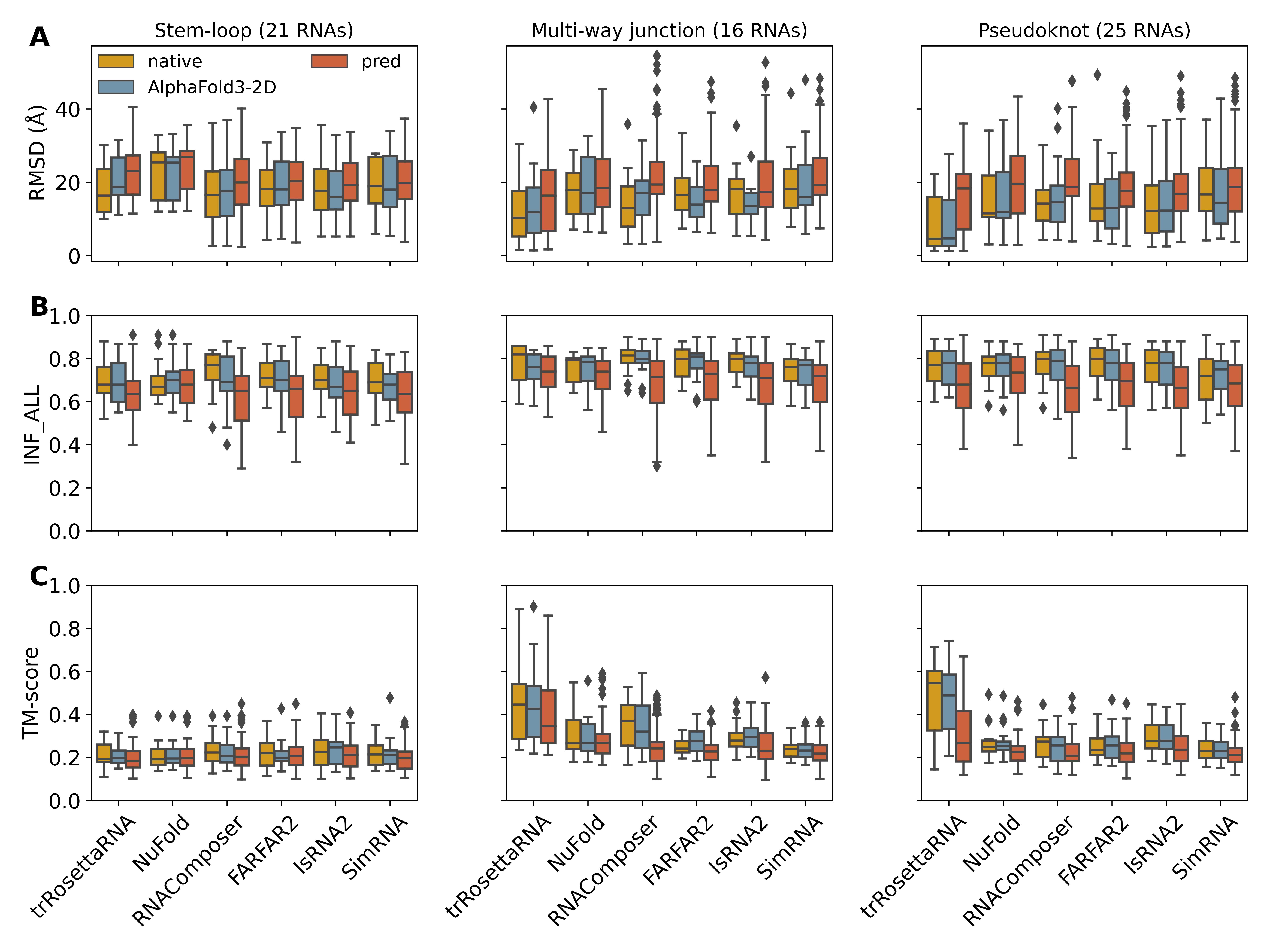


**Fig S4.** Box plots of (A) RMSD, (B) $INF\_ALL$, and (C) TMscore for predictions from five tested 3D models using different 2D structures as input: stem-loops (first column), multi-way junctions (second column), and pseudoknots (third column). Predictions using native 2D structures and 2D structures derived from 3D structures predicted by AlphaFold3 as inputs are denoted as “native” and “AlphaFold3”, respectively, while “pred” represents the values of 3D structure predictions using different 2D structures generated by the six selected 2D tools as input. The whiskers of each box plot extend to the most extreme data points within 1.5 times the interquartile range (IQR) from the lower and upper quartiles, respectively. Observations beyond this range are plotted individually as outliers (grey diamonds).


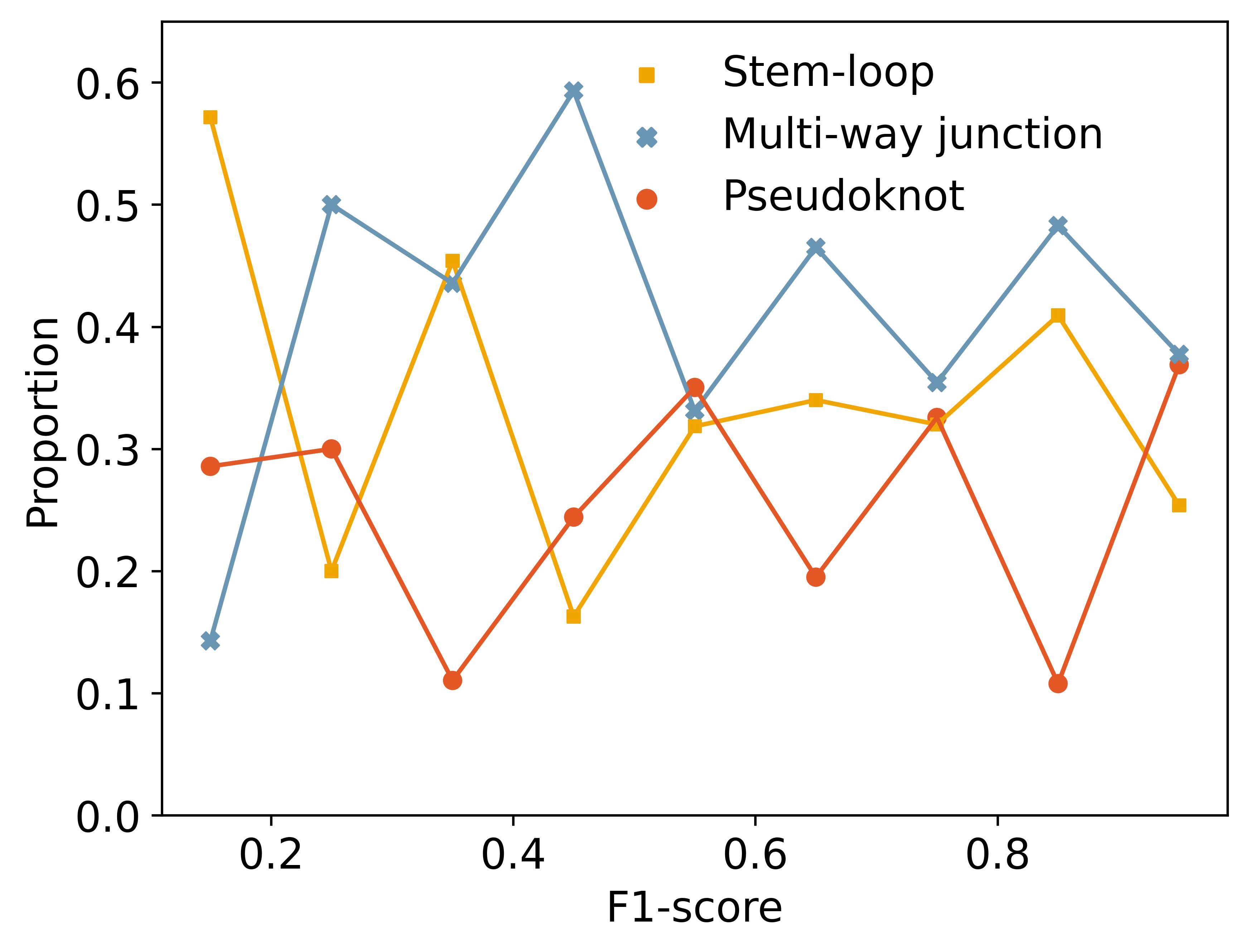


**Fig S5.** The proportion of different topological categories as functions of the F1-score values of input 2D structures. The F1-score values were grouped by a bin size of 0.1. Data represent the mean value of a particular bin.


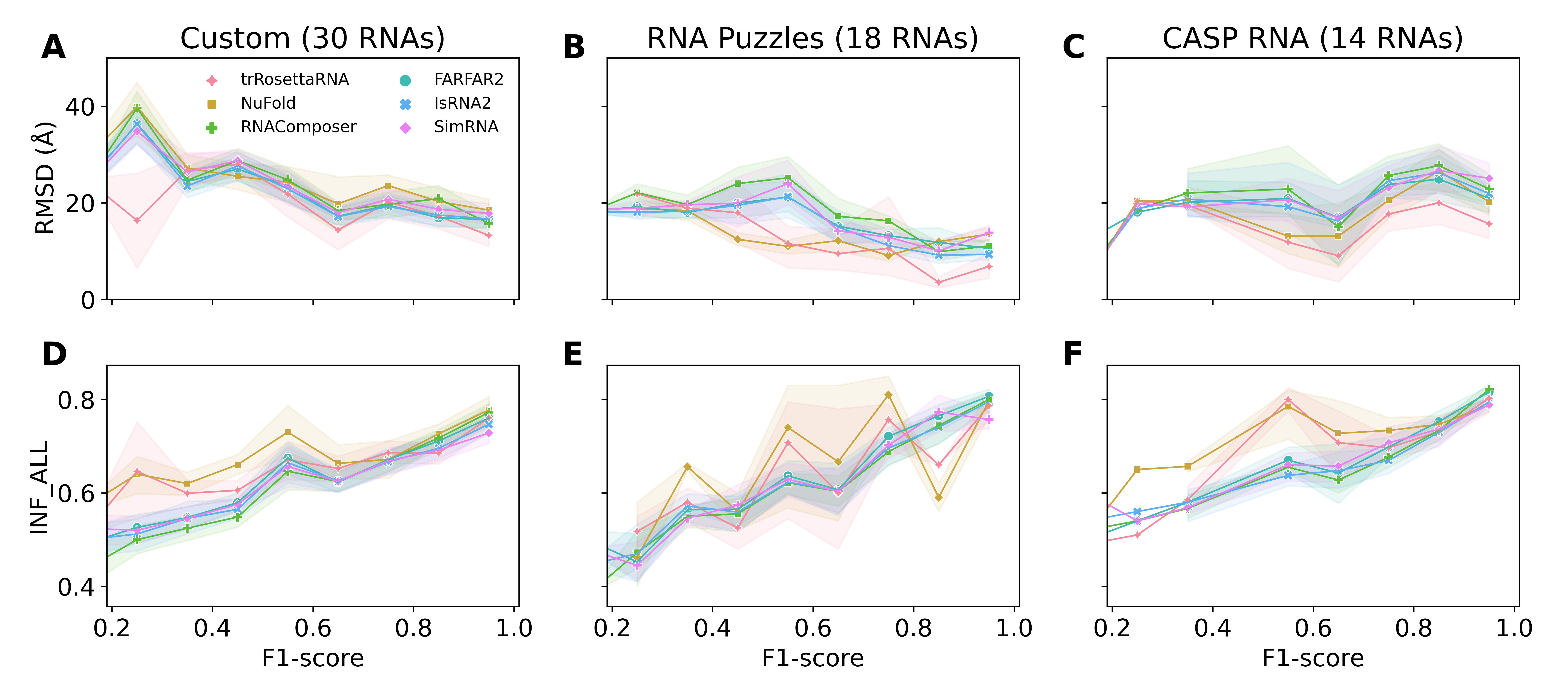


**Fig S6.** Detailed relationship between the (A-C) RMSD and (D-F) INF_ALL values of RNA 3D structures predicted by the five selected models and the F1-score values of input 2D structures on different test datasets: (A, D) *Custom* dataset, (B, E) *RNA Puzzles*, and (C, F) *CASP RNA*. The F1-score values were grouped by a bin size of 0.1. Symbols represent the mean metrics of a particular bin and shaded area indicates the 95% confidence interval. The analysis considered all 2D structures predicted by the selected six 2D tools, AlphaFold3-derived 2D structures, and the native 2D structures.


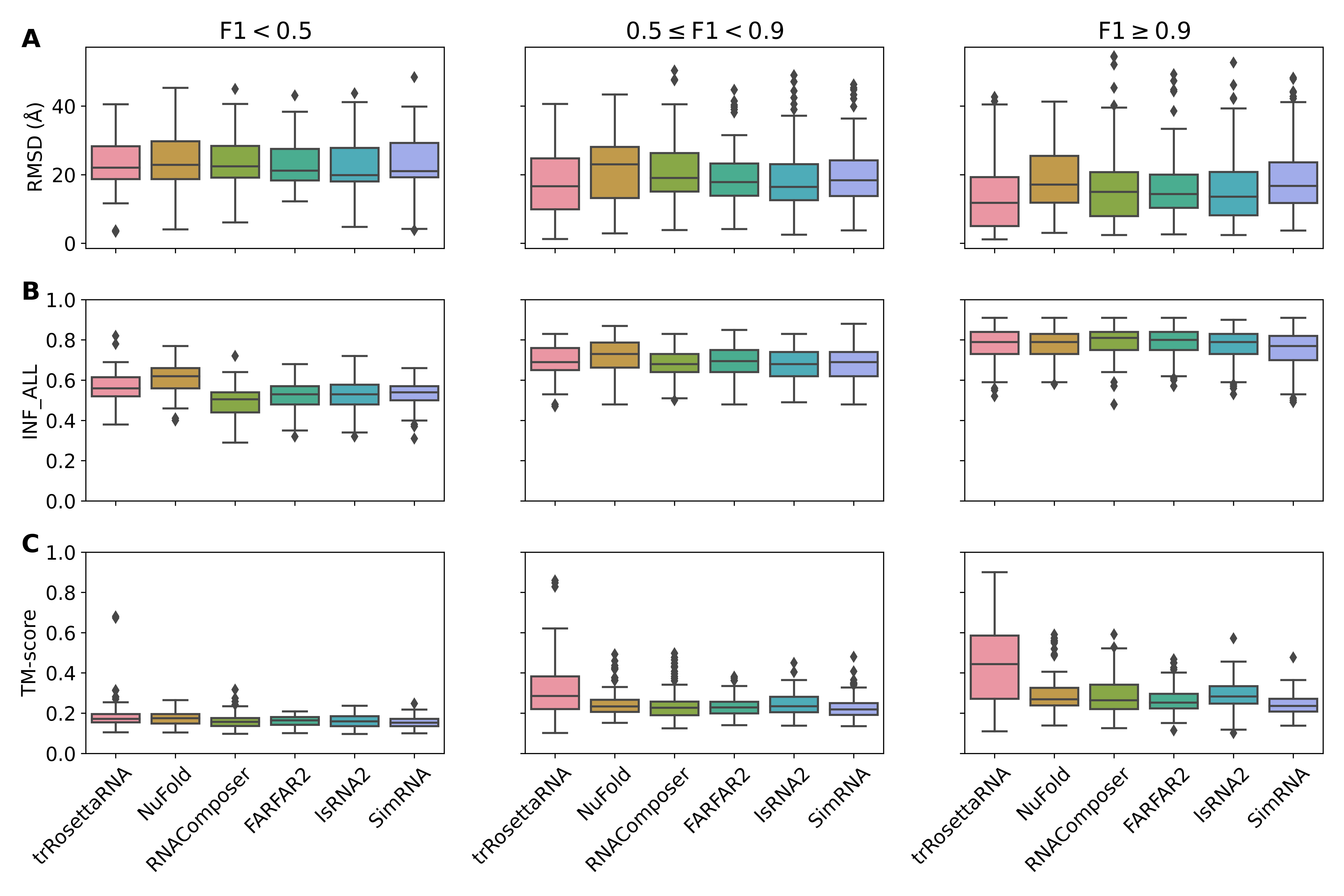


**Fig S7.** Accurate 2D structures as input can largely improve the performance of RNA 3D structure prediction. Box plots of (A) RMSD, (B) INF_ALL, and (C) TMscore values of RNA 3D structure predictions based on different accuracy levels of input 2D structures: low accuracy ($\text{F1-score}<0.5$), medium accuracy ($0.5\leq\text{F1-score}<0.9$), and high accuracy ($\text{F1-score}\geq0.9$). The whiskers of each box plot extend to the most extreme data points within 1.5 times the interquartile range (IQR) from the lower and upper quartiles, respectively. Observations beyond this range are plotted individually as outliers (grey diamonds).





**Fig S8.** Scatter plots shown the relationships between the (A) proportion of true positive ($\alpha_{TP}$) base pairs and (B) proportion of false positive ($\alpha_{FP}$) base pairs in the input 2D structure and the RMSD values of 3D structures predicted by different 3D models. The line in each plane represents the linear fitting result, and the corresponding Pearson correlation coefficient (*R*) and *p*-value (*p*) are also presented. From left to right: trRosettaRNA, NuFold, RNAComposer, FARFAR2, IsRNA2, and SimRNA.





**Fig S9.** Scatter plots shown the relationships between the (A) RMSD, (B) INF_ALL and (C) TMscore and the F1-score of input 2D structures predicted by different 2D models for target R1108 from the *CASP RNA* dataset. The line in each plot represents the linear fitting result, and the corresponding Pearson correlation coefficient (*R*) and *p*-value (*p*) are also presented. From left to right: NuFold, trRosettaRNA, RNAComposer, FARFAR2, IsRNA2, and SimRNA.


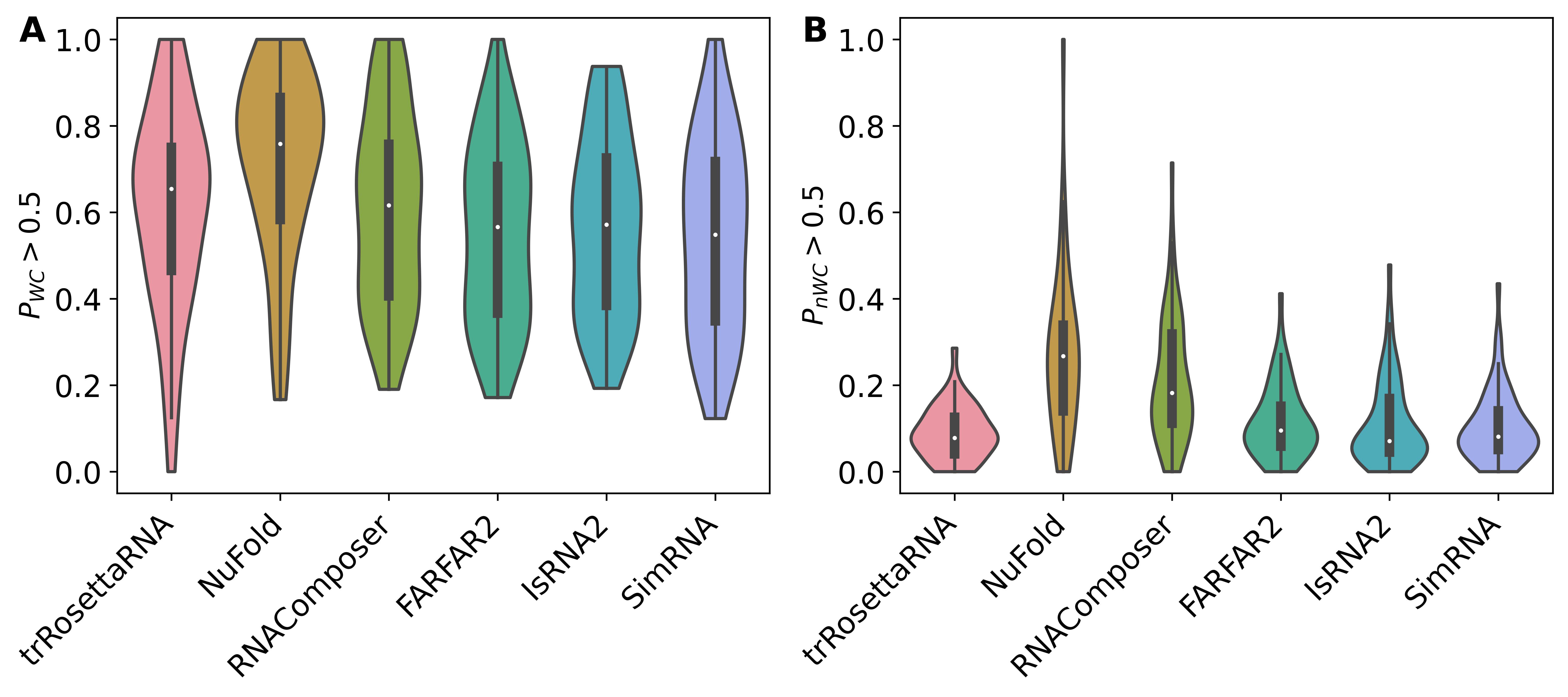


**Fig S10.** Violin plots of the distribution of the proportion of (A) Watson-Crick (WC) and (B) non-Watson-Crick (nWC) base pairs that were retained in more than 50% of the tertiary structures predicted from the various predicted 2D structure for various RNA 3D structure prediction models. The proportion was calculated across all associated 3D prediction for each target RNA in the Combined dataset.


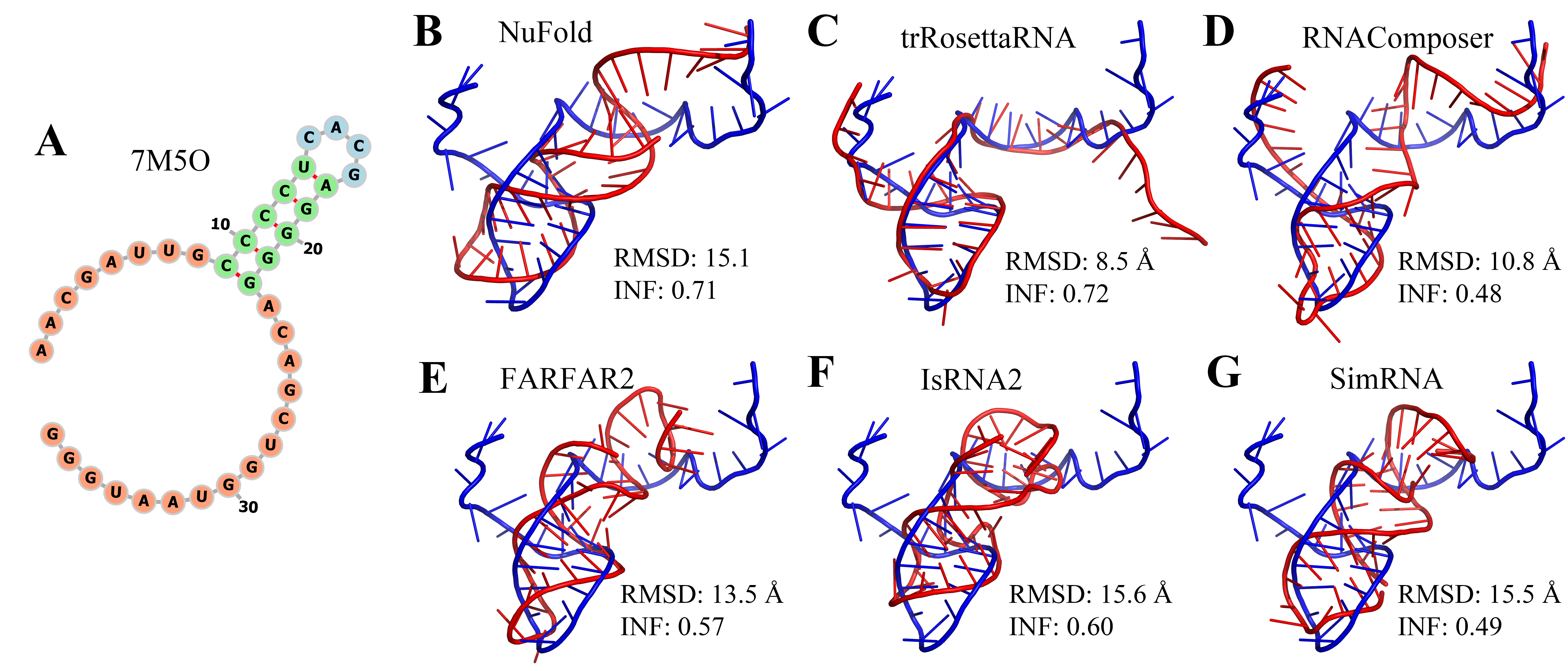


**Fig S11.** (A) Native 2D structure of the crRNA in the CRISPR-Cas Phi system (PDB id: 7M5O [44]). Using the native 2D structure as input, the 3D structures predicted by different 3D models: (B) NuFold, (C) trRosettaRNA, (D) RNAComposer, (E) FARFAR2, (F) IsRNA2, and (G) SimRNA. INF_ALL and RMSD values between the predicted (in red) and native (in blue) structures for each model are also shown.


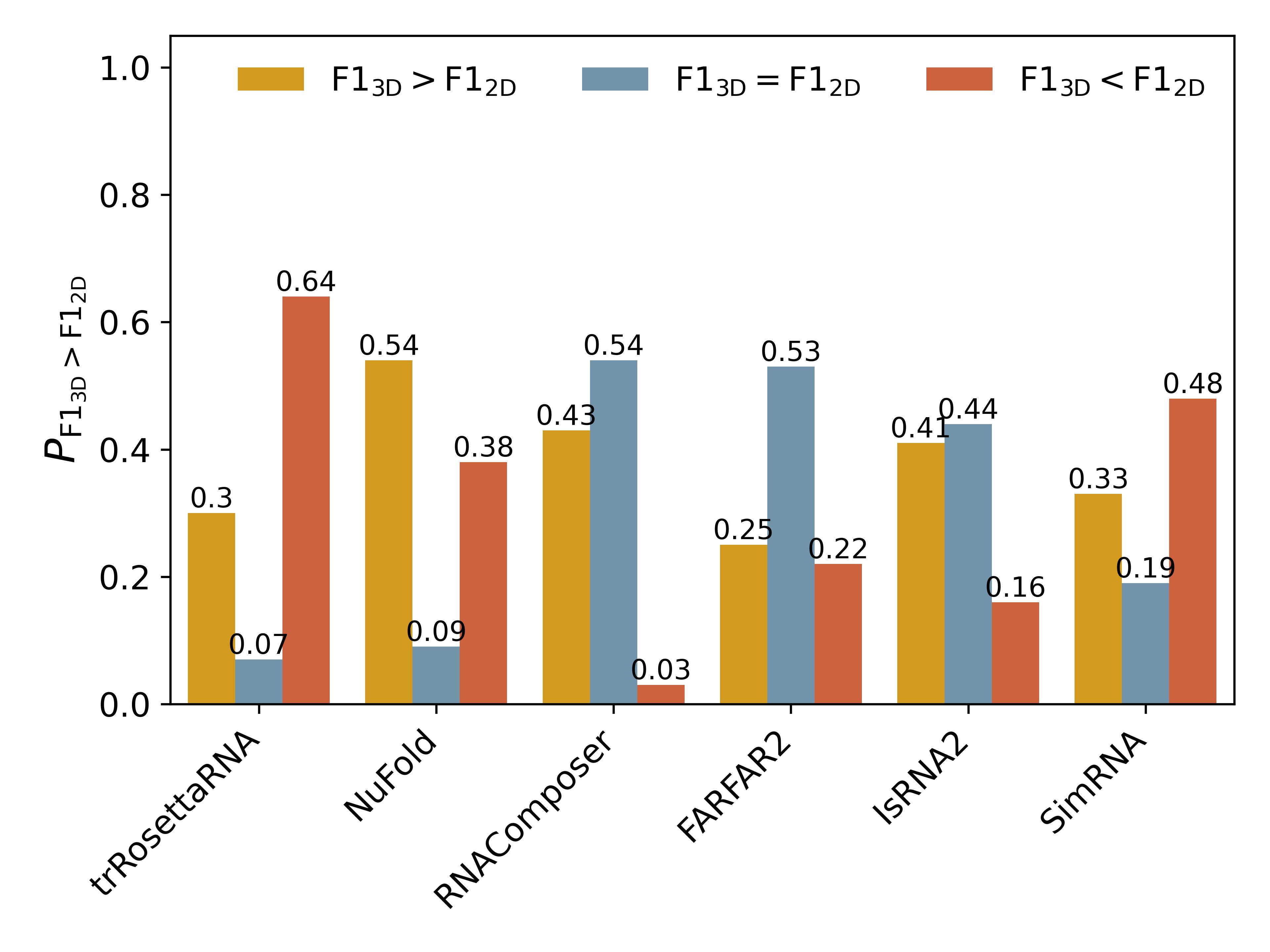


**Fig S12.** Histogram of the fraction of F1-score after 3D structure prediction (${F1}_{3D}$) greater than, equal to, and lower than the F1-score of the input 2D structure (${F1}_{2D}$) for each tested 3D model. Here all the combination of selected six 2D and five 3D methods were considered.

## S3. Supplementary Tables

**Table S1 Detailed information of 2D tools**

| **2D tools** | **Version** | **URL** | **Run Command** |
| --- | --- | --- | --- |
| RNAfold | v2.6.4 | http://www.tbi.univie.ac.at/RNA/ | <ViennaRNA_PATH>/src/bin/RNAfold <fasta_file> |
| RNAStructure | v6.4 | https://rna.urmc.rochester.edu/RNAstructure.html | export <RNAStructure_PATH>/data_tables/ && <RNAStructure_PATH>/exe/Fold <fasta_file> <output_file> |
| CONTRAfold | v2.02 | http://contra.stanford.edu/contrafold/ | <CONTRAfold_PATH>/src/contrafold predict <fasta_file> |
| Mfold | v3.6 | https://www.unafold.org/mfold/software/download-mfold.php | <mfold_PATH> SEQ=<seq_file> |
| NUPACK | v4.0 | https://nupack.org/download/overview | Python code:  *Set1 = nupack.ComplexSet(strands=[nupack.Strand(seqfile, seq_id)], complexes=SetSpec(max_size=1)); run_res = nupack.complex_analysis(Set1, Model(), compute=['mfe']); out_ss = list(run_res.as_dict()['complexes'].values())[0].mfe[0].structure.dotparensplus();* |
| MXfold2 | - | https://github.com/mxfold/mxfold2 | mxfold2 predict <fasta_file> |
| SPOT-RNA | - | https://github.com/jaswindersingh2/SPOT-RNA | python <SPOT_RNA_PATH>/SPOT-RNA.py --inputs <fasta_file> --outputs <out_dir> |
| Ufold | - | http://146.56.237.198:3838/UFold/ | Webserver with default settings. |
| CentroidFold | v0.0.16 | http://rtools.cbrc.jp/centroidfold/ | Webserver with default settings. |
| PETfold | v1.0 | https://rth.dk/resources/petfold/download.php | export <RNAfold_bin_PATH> && export <PETfold1_PATH>/programs/pfold/bin && <PETfold1_PATH>/bin/PETfold.pl -f <fasta_file> |
| PETfold | v2.2 | https://rth.dk/resources/petfold/download.php | <PETfold2_PATH>/bin/PETfold -f <fasta_file> |
| BPfold | - | https://github.com/heqin-zhu/BPfold | BPfold --checkpoint_dir <checkpoint> --input <fasta_file> --output <out_dir> |
| LinearFold | - | https://github.com/LinearFold/LinearFold | echo <seq_string> \| <LinearFold_PATH>/linearfold |
| EternaFold | v.1.3.1 | https://github.com/eternagame/EternaFold | <EternaFold_PATH>/src/contrafold predict <fasta_file> --params <param_file> |

**Table S2. Commands to run 3D models**

| **3D Model** | **Run Command** | **No. of available outputs**  **(per run task)** |
| --- | --- | --- |
| trRosettaRNA | python predict.py -i <msa_file> -ss <ss_file> -ss_fmt dot_bracket -o <npz_file> -mdir <model_path> -gpu 0 && python fold.py -npz <npz_file> -fa <fasta_file> -o <pdb_file_name> -cpu 32 | 1 |
| DRfold | bash DRfold.sh <fasta_file> <output_dir> | 1 |
| NuFold | python run_nufold.py –ckpt_path <model_path> --input_fasta <fasta_file> --input_dir <input_dir (contains fasta, MSA, and 2D structure file)> --ouput_dir <output_dir> --config_preset initial_training | 4 |
| SimRNA | SimRNA -s <fasta_file> -S <ss_file> -c <config_file> && clustering <trafl_output_file> 0.01 3.5 5.0 | More than 1  (Not fixed) |
| IsRNA2 | IsRNA-Tertiary_Folding_NP.out <Input_Data> <ss_file> <work_dir> <jobname> <config_file> 0 <excluded_pdblist_file> 1 0 | 5  (user-configurable) |
| FARFAR2 | rna_denovo.linuxgccrelease -sequence <seq_string> -secstruct <ss_string> -nstruct <#output> -out:path <output_dir> -minimize_rna | 5  (user-configurable) |
| AlphaFold3 | The webserver was used with default settings. | 5  (user-configurable) |
| RNAComposer | The webserver was used with default settings and the native PDB file of each input was uploaded to active the 'exclude PDB' option. | 5  (user-configurable) |

**Table S3. List of RNAs in the *Custom* dataset.**

| **PDB ID** | **Chain ID** | **Structure type** | **Sequence length** | **No. of WC pairs** |
| --- | --- | --- | --- | --- |
| 7DLZ | X | stem-loop | 45 | 15 |
| 7DVQ | F | stem-loop | 64 | 12 |
| 7DVQ | H | stem-loop | 51 | 6 |
| 7EOG | A | stem-loop | 48 | 15 |
| 7KVU | G | pseudoknot | 82 | 23 |
| 7LJ3 | A | multi-way junction | 75 | 21 |
| 7M5O | B | stem-loop | 37 | 5 |
| 7MLW | F | pseudoknot | 129 | 40 |
| 7PKQ | 1 | stem-loop | 97 | 7 |
| 7PKT | 4 | stem-loop | 118 | 17 |
| 7PKT | 5 | multi-way junction | 111 | 19 |
| 7PKT | 6 | stem-loop | 136 | 13 |
| 7PKT | 2 | stem-loop | 86 | 10 |
| 7PKT | 3 | multi-way junction | 184 | 39 |
| 7R6Q | 1 | multi-way junction | 93 | 22 |
| 7UMC | A | multi-way junction | 70 | 21 |
| 7UTN | C | pseudoknot | 192 | 52 |
| 8BTZ | A | pseudoknot | 238 | 107 |
| 8DZJ | E | pseudoknot | 157 | 43 |
| 8EUG | 9 | multi-way junction | 118 | 37 |
| 8FFY | C | pseudoknot | 65 | 20 |
| 8HMZ | 3 | stem-loop | 37 | 5 |
| 8HMZ | 5 | stem-loop | 48 | 7 |
| 8PNQ | V | stem-loop | 30 | 2 |
| 8Y6O | K | stem-loop | 48 | 6 |
| 8YDC | A | pseudoknot | 57 | 10 |
| 9BH5 | A8 | pseudoknot | 150 | 24 |
| 9CES | W | stem-loop | 142 | 33 |
| 9CET | W | stem-loop | 54 | 15 |
| 9CF3 | W | stem-loop | 44 | 9 |

**Table S4. List of RNAs in the *RNA Puzzles* dataset.**

| **Puzzle ID** | **Structure type** | **Sequence length** | **No. of WC pairs** |
| --- | --- | --- | --- |
| PZ5 | multi-way junction | 197 | 60 |
| PZ10 | pseudoknot | 99 | 27 |
| PZ13 | stem-loop | 64 | 19 |
| PZ14 | pseudoknot | 60 | 17 |
| PZ15 | multi-way junction | 72 | 17 |
| PZ17 | multi-way junction | 62 | 19 |
| PZ21 | pseudoknot | 39 | 10 |
| PZ29 | pseudoknot | 53 | 20 |
| PZ30 | stem-loop | 97 | 27 |
| PZ31 | pseudoknot | 65 | 23 |
| PZ32 | stem-loop | 49 | 16 |
| PZ33 | pseudoknot | 45 | 15 |
| PZ34 | multi-way junction | 68 | 20 |
| PZ35 | pseudoknot | 69 | 21 |
| PZ36 | pseudoknot | 69 | 22 |
| PZ37 | pseudoknot | 61 | 14 |
| PZ38 | pseudoknot | 55 | 13 |
| PZ39 | multi-way junction | 90 | 35 |

**Table S5. List of RNAs in the *CASP RNA* dataset.**

| **CASP RNA ID** | **Structure type** | **Sequence length** | **No. of WC pairs** |
| --- | --- | --- | --- |
| R1107 | pseudoknot | 69 | 21 |
| R1108 | pseudoknot | 69 | 22 |
| R1116 | multi-way junction | 150 | 51 |
| R1117 | pseudoknot | 30 | 8 |
| R1126 | multi-way junction | 363 | 145 |
| R1128 | pseudoknot | 238 | 107 |
| R1136 | pseudoknot | 374 | 153 |
| R1149 | multi-way junction | 124 | 44 |
| R1156 | multi-way junction | 135 | 47 |
| R1189 | stem-loop | 118 | 25 |
| R1190 | stem-loop | 118 | 33 |
| R1203 | multi-way junction | 134 | 44 |
| R1212 | pseudoknot | 125 | 42 |
| R1261 | pseudoknot | 89 | 29 |

**Table S6. More detailed info of RNAs in our test datasets.**

| **Case ID** | **PDB ID** | **Release**  **Date** | **Resolution**  **(Å)** | **in**  ***NuFold***  **Dataset**  **(before**  **28/2/2022)** | **in**  ***TrRosetta-RNA***  **Dataset**  **(before**  **1/1/2022)** | **in**  ***Alpha-Fold3***  **Dataset**  **(before**  **12/1/2023)** | **in**  ***DRfold***  **Dataset**  **(before**  **1/1/2021)** | **Dataset**  **category** |
| --- | --- | --- | --- | --- | --- | --- | --- | --- |
| R1138 | 7PTK,  7PTL | 10/5/2022 | 5.18 | No | no | yes | no | *CASP RNA* |
| R1107 | 7QR4 | 10/26/2022 | 2.83 | no | no | yes | no | *CASP RNA* |
| R1108 | 7QR3 | 10/26/2022 | 2.18 | no | no | yes | no | *CASP RNA* |
| R1128 | 8BTZ | 12/28/2022 | 5.39 | no | no | yes | no | *CASP RNA* |
| R1136 | 7ZJ4 | 4/19/2023 | 4.43 | no | no | no | no | *CASP RNA* |
| R1189 | 7YR7 | 5/17/2023 | 3.8 | no | no | no | no | *CASP RNA* |
| R1190 | 7YR6 | 5/17/2023 | 4.8 | no | no | no | no | *CASP RNA* |
| R1116 | 8S95 | 8/9/2023 | 3.1 | no | no | no | no | *CASP RNA* |
| R1117 | 8FZA | 8/30/2023 | 2.3 | no | no | no | no | *CASP RNA* |
| R1149 | 8UYS | 12/6/2023 | 4.7 | no | no | no | no | *CASP RNA* |
| R1156 | 8UYE,  8UYG,  8UYJ | 12/6/2023 | 5.9 | no | no | no | no | *CASP RNA* |
| R1126 | 8TVZ | 4/3/2024 | 5.94 | no | no | no | no | *CASP RNA* |
| R1203 | 8UO6 | 6/5/2024 | 2.85 | no | no | no | no | *CASP RNA* |
| R1212 | 9B0L | 10/9/2024 | 2.99 | no | no | no | no | *CASP RNA* |
| R1261 | 9BZC | 11/27/2024 | 2.52 | no | no | no | no | *CASP RNA* |
| R1262 | 9BZ1 | 11/27/2024 | 2.8 | no | no | no | no | *CASP RNA* |
| 7DVQ-H | 7DVQ | 3/31/2021 | 2.89 | yes | yes | yes | no | *Custom* |
| 7DVQ-F | 7DVQ | 3/31/2021 | 2.89 | yes | yes | yes | no | *Custom* |
| 7M5O-B | 7M5O | 8/4/2021 | 3.54 | yes | yes | yes | no | *Custom* |
| 7DLZ-X | 7DLZ | 10/27/2021 | 3 | yes | yes | yes | no | *Custom* |
| 7LJ3-A | 7LJ3 | 11/17/2021 | 2.9 | yes | yes | yes | no | *Custom* |
| 7EOG-A | 7EOG | 11/24/2021 | 1.5 | yes | yes | yes | no | *Custom* |
| 7MLW-F | 7MLW | 1/12/2022 | 2.7 | yes | no | yes | no | *Custom* |
| 7KVU-G | 7KVU | 1/19/2022 | 2.68 | yes | no | yes | no | *Custom* |
| 7PKT-3 | 7PKT | 6/15/2022 | 3 | no | no | yes | no | *Custom* |
| 7PKT-2 | 7PKT | 6/15/2022 | 3 | no | no | yes | no | *Custom* |
| 7PKT-6 | 7PKT | 6/15/2022 | 3 | no | no | yes | no | *Custom* |
| 7PKT-5 | 7PKT | 6/15/2022 | 3 | no | no | yes | no | *Custom* |
| 7UTN-C | 7UTN | 6/15/2022 | 2.74 | no | no | yes | no | *Custom* |
| 7PKT-4 | 7PKT | 6/15/2022 | 3 | no | no | yes | no | *Custom* |
| 7UMC-A | 7UMC | 7/6/2022 |  | no | no | yes | no | *Custom* |
| 7R6Q-1 | 7R6Q | 11/9/2022 | 2.98 | no | no | yes | no | *Custom* |
| 8EUG-9 | 8EUG | 11/30/2022 | 2.8 | no | no | yes | no | *Custom* |
| 8BTZ-A | 8BTZ | 12/28/2022 | 5.39 | no | no | yes | no | *Custom* |
| 8HMZ-3 | 8HMZ | 4/19/2023 | 2.9 | no | no | no | no | *Custom* |
| 8HMZ-5 | 8HMZ | 4/19/2023 | 2.9 | no | no | no | no | *Custom* |
| 7PKQ-1 | 7PKQ | 6/14/2023 | 4.2 | no | no | no | no | *Custom* |
| 8DZJ-E | 8DZJ | 7/12/2023 | 2.9 | no | no | no | no | *Custom* |
| 8FFY-C | 8FFY | 8/23/2023 | 3.6 | no | no | no | no | *Custom* |
| 8PNQ-V | 8PNQ | 2/21/2024 | 2.88 | no | no | no | no | *Custom* |
| 8Y6O-K | 8Y6O | 3/20/2024 | 3.38 | no | no | no | no | *Custom* |
| 8YDC-A | 8YDC | 8/21/2024 | 2.89 | no | no | no | no | *Custom* |
| 9BH5-A8 | 9BH5 | 9/4/2024 | 2.63 | no | no | no | no | *Custom* |
| 9CES-W | 9CES | 9/11/2024 | 3.28 | no | no | no | no | *Custom* |
| 9CET-W | 9CET | 9/11/2024 | 3 | no | no | no | no | *Custom* |
| 9CF3-W | 9CF3 | 9/11/2024 | 3.2 | no | no | no | no | *Custom* |
| PZ5 | 4P9R | 5/28/2014 | 2.7 | yes | yes | yes | yes | *RNA Puzzles* |
| PZ10 | 4LCK | 7/31/2013 | 3.2 | yes | yes | yes | yes | *RNA Puzzles* |
| PZ13 | 4XW7 | 9/9/2015 | 2.5 | yes | yes | yes | yes | *RNA Puzzles* |
| PZ14 | 5DDO | 12/23/2015 | 3.1 | yes | yes | yes | yes | *RNA Puzzles* |
| PZ15 | 5DI4 | 10/7/2015 | 2.95 | yes | yes | yes | yes | *RNA Puzzles* |
| PZ17 | 5K7C | 7/13/2016 | 2.73 | yes | yes | yes | yes | *RNA Puzzles* |
| PZ21 | 5NWQ,  5NZ6 | 10/18/2017 | 1.91 | yes | yes | yes | yes | *RNA Puzzles* |
| PZ29 | 6TB7 | 9/30/2020 | 2.53 | yes | yes | yes | yes | *RNA Puzzles* |
| PZ30 | 7BG9 | 4/28/2021 | 3.8 | yes | yes | yes | no | *RNA Puzzles* |
| PZ33 | 7ELP,  7ELQ,  7ELR,  7ELS | 6/30/2021 | 2.79 | yes | yes | yes | no | *RNA Puzzles* |
| PZ31 | 7MLX | 8/11/2021 | 2.09 | yes | yes | yes | no | *RNA Puzzles* |
| PZ32 | 7EOJ | 11/24/2021 | 1.77 | yes | yes | yes | no | *RNA Puzzles* |
| PZ34 | 7V9E | 3/23/2022 | 2.3 | no | no | yes | no | *RNA Puzzles* |
| PZ36 | 7QR3 | 10/26/2022 | 2.18 | no | no | yes | no | *RNA Puzzles* |
| PZ35 | 7QR4 | 10/26/2022 | 2.83 | no | no | yes | no | *RNA Puzzles* |
| PZ37 | 8GXC | 1/18/2023 | 2.5 | no | no | no | no | *RNA Puzzles* |
| PZ38 | 8HB8 | 3/22/2023 | 2.3 | no | no | no | no | *RNA Puzzles* |
| PZ39 | 8DP3 | 4/19/2023 | 1.91 | no | no | no | no | *RNA Puzzles* |

# References

[1] Abramson J, Adler J, Dunger J, Evans R, Green T, Pritzel A, et al. Accurate structure prediction of biomolecular interactions with AlphaFold 3. Nature 2024;630:493–500. https://doi.org/10.1038/s41586-024-07487-w.

[2] Wang W, Feng C, Han R, Wang Z, Ye L, Du Z, et al. trRosettaRNA: automated prediction of RNA 3D structure with transformer network. Nat Commun 2023;14:7266. https://doi.org/10.1038/s41467-023-42528-4.

[3] Das R, Kretsch RC, Simpkin AJ, Mulvaney T, Pham P, Rangan R, et al. Assessment of three‐dimensional RNA structure prediction in CASP15. Proteins 2023;91:1747–70. https://doi.org/10.1002/prot.26602.

[4] Li Y, Zhang C, Feng C, Pearce R, Lydia Freddolino P, Zhang Y. Integrating end-to-end learning with deep geometrical potentials for ab initio RNA structure prediction. Nat Commun 2023;14:5745. https://doi.org/10.1038/s41467-023-41303-9.

[5] Perry ZR, Pyle AM, Zhang C. Arena: Rapid and Accurate Reconstruction of Full Atomic RNA Structures From Coarse-grained Models. J Mol Biol 2023;435:168210.

[6] Eastman P, Swails J, Chodera JD, McGibbon RT, Zhao Y, Beauchamp KA, et al. OpenMM 7: Rapid development of high performance algorithms for molecular dynamics. PLoS Comput Biol 2017;13:e1005659.

[7] Kagaya Y, Zhang Z, Ibtehaz N, Wang X, Nakamura T, Punuru PD, et al. NuFold: end-to-end approach for RNA tertiary structure prediction with flexible nucleobase center representation. Nat Commun 2025;16:881. https://doi.org/10.1038/s41467-025-56261-7.

[8] Popenda M, Szachniuk M, Antczak M, Purzycka KJ, Lukasiak P, Bartol N, et al. Automated 3D structure composition for large RNAs. Nucleic Acids Res 2012;40:e112–e112.

[9] Sarzynska J, Popenda M, Antczak M, Szachniuk M. RNA tertiary structure prediction using RNAComposer in CASP15. Proteins 2023;91:1790–9. https://doi.org/10.1002/prot.26578.

[10] Popenda M, Szachniuk M, Blazewicz M, Wasik S, Burke EK, Blazewicz J, et al. RNA FRABASE 2.0: an advanced web-accessible database with the capacity to search the three-dimensional fragments within RNA structures. BMC Bioinformatics 2010;11:1–12.

[11] Watkins AM, Rangan R, Das R. FARFAR2: improved de novo rosetta prediction of complex global RNA folds. Structure 2020;28:963–76.

[12] Das R, Baker D. Automated de novo prediction of native-like RNA tertiary structures. Proc Natl Acad Sci 2007;104:14664–9.

[13] Leontis NB, Zirbel CL. Nonredundant 3D structure datasets for RNA knowledge extraction and benchmarking. RNA 3D Struct Anal Predict 2012:281–98.

[14] Lyskov S, Chou F-C, Conchuir SO, Der BS, Drew K, Kuroda D, et al. Serverification of molecular modeling applications: the Rosetta Online Server that Includes Everyone (ROSIE). PloS One 2013;8:e63906.

[15] Moretti R, Lyskov S, Das R, Meiler J, Gray JJ. Web-accessible molecular modeling with Rosetta: the Rosetta online server that includes everyone (ROSIE). Protein Sci 2018;27:259–68.

[16] Zhang D, Chen S-J, Zhou R. Modeling Noncanonical RNA Base Pairs by a Coarse-Grained IsRNA2 Model. J Phys Chem B 2021;125:11907–15. https://doi.org/10.1021/acs.jpcb.1c07288.

[17] Zhang D, Chen S-J. IsRNA: An iterative simulated reference state approach to modeling correlated interactions in RNA folding. J Chem Theory Comput 2018;14:2230–9.

[18] Zhang D, Li J, Chen S-J. IsRNA1: de novo prediction and blind screening of RNA 3D structures. J Chem Theory Comput 2021;17:1842–57.

[19] Boniecki MJ, Lach G, Dawson WK, Tomala K, Lukasz P, Soltysinski T, et al. SimRNA: a coarse-grained method for RNA folding simulations and 3D structure prediction. Nucleic Acids Res 2016;44:e63–e63. https://doi.org/10.1093/nar/gkv1479.

[20] Lorenz R, Bernhart SH, Höner zu Siederdissen C, Tafer H, Flamm C, Stadler PF, et al. ViennaRNA Package 2.0. Algorithms Mol Biol 2011;6:1–14. https://doi.org/10.1186/1748-7188-6-26.

[21] Reuter JS, Mathews DH. RNAstructure: software for RNA secondary structure prediction and analysis. BMC Bioinformatics 2010;11:1–9. https://doi.org/10.1186/1471-2105-11-129.

[22] Do CB, Woods DA, Batzoglou S. CONTRAfold: RNA secondary structure prediction without physics-based models. Bioinformatics 2006;22:e90–8. https://doi.org/10.1093/bioinformatics/btl246.

[23] Zuker M. Mfold web server for nucleic acid folding and hybridization prediction. Nucleic Acids Res 2003;31:3406–15. https://doi.org/10.1093/nar/gkg595.

[24] Zuker M, Mathews DH, Turner DH. Algorithms and thermodynamics for RNA secondary structure prediction: a practical guide. RNA Biochem Biotechnol 1999:11–43. https://doi.org/10.1007/978-94-011-4485-8_2.

[25] Rouillard J-M, Zuker M, Gulari E. OligoArray 2.0: design of oligonucleotide probes for DNA microarrays using a thermodynamic approach. Nucleic Acids Res 2003;31:3057–62. https://doi.org/10.1093/nar/gkg426.

[26] Zadeh JN, Steenberg CD, Bois JS, Wolfe BR, Pierce MB, Khan AR, et al. NUPACK: Analysis and design of nucleic acid systems. J Comput Chem 2011;32:170–3. https://doi.org/10.1002/jcc.21596.

[27] Sato K, Akiyama M, Sakakibara Y. RNA secondary structure prediction using deep learning with thermodynamic integration. Nat Commun 2021;12:941.

[28] Singh J, Hanson J, Paliwal K, Zhou Y. RNA secondary structure prediction using an ensemble of two-dimensional deep neural networks and transfer learning. Nat Commun 2019;10:5407. https://doi.org/10.1038/s41467-019-13395-9.

[29] Fu L, Cao Y, Wu J, Peng Q, Nie Q, Xie X. UFold: fast and accurate RNA secondary structure prediction with deep learning. Nucleic Acids Res 2022;50:e14–e14. https://doi.org/10.1093/nar/gkab1074.

[30] Hamada M, Yamada K, Sato K, Frith MC, Asai K. CentroidHomfold-LAST: accurate prediction of RNA secondary structure using automatically collected homologous sequences. Nucleic Acids Res 2011;39:W100–6. https://doi.org/10.1093/nar/gkr290.

[31] Seemann SE, Gorodkin J, Backofen R. Unifying evolutionary and thermodynamic information for RNA folding of multiple alignments. Nucleic Acids Res 2008;36:6355–62. https://doi.org/10.1093/nar/gkn544.

[32] Zhu H, Tang F, Quan Q, Chen K, Xiong P, Zhou SK. Deep generalizable prediction of RNA secondary structure via base pair motif energy. Nat Commun 2025;16:5856. https://doi.org/10.1038/s41467-025-60048-1.

[33] Huang L, Zhang H, Deng D, Zhao K, Liu K, Hendrix DA, et al. LinearFold: linear-time approximate RNA folding by 5’-to-3’dynamic programming and beam search. Bioinformatics 2019;35:i295–304.

[34] Wayment-Steele HK, Kladwang W, Strom AI, Lee J, Treuille A, Becka A, et al. RNA secondary structure packages evaluated and improved by high-throughput experiments. Nat Methods 2022;19:1234–42. https://doi.org/10.1038/s41592-022-01605-0.

[35] Magnus M, Antczak M, Zok T, Wiedemann J, Lukasiak P, Cao Y, et al. RNA-Puzzles toolkit: a computational resource of RNA 3D structure benchmark datasets, structure manipulation, and evaluation tools. Nucleic Acids Res 2020;48:576–88.

[36] Zhang Y, Skolnick J. Scoring function for automated assessment of protein structure template quality. Proteins 2004;57:702–10.

[37] Gong S, Zhang C, Zhang Y. RNA-align: quick and accurate alignment of RNA 3D structures based on size-independent TM-scoreRNA. Bioinformatics 2019;35:4459–61.

[38] Zhang C, Shine M, Pyle AM, Zhang Y. US-align: universal structure alignments of proteins, nucleic acids, and macromolecular complexes. Nat Methods 2022;19:1109–15.

[39] Mariani V, Biasini M, Barbato A, Schwede T. lDDT: a local superposition-free score for comparing protein structures and models using distance difference tests. Bioinformatics 2013;29:2722–8. https://doi.org/10.1093/bioinformatics/btt473.

[40] Biasini M, Mariani V, Haas J, Scheuber S, Schenk AD, Schwede T, et al. OpenStructure: a flexible software framework for computational structural biology. Bioinformatics 2010;26:2626–8.

[41] Lu X-J, Bussemaker HJ, Olson WK. DSSR: an integrated software tool for dissecting the spatial structure of RNA. Nucleic Acids Res 2015;43:e142–e142. https://doi.org/10.1093/nar/gkv716.

[42] Yang H, Jossinet F, Leontis N, Chen L, Westbrook J, Berman H, et al. Tools for the automatic identification and classification of RNA base pairs. Nucleic Acids Res 2003;31:3450–60. https://doi.org/10.1093/nar/gkg529.

[43] Gendron P, Lemieux S, Major F. Quantitative analysis of nucleic acid three-dimensional structures. J Mol Biol 2001;308:919–36. https://doi.org/10.1006/jmbi.2001.4626.

[44] Pausch P, Soczek KM, Herbst DA, Tsuchida CA, Al-Shayeb B, Banfield JF, et al. DNA interference states of the hypercompact CRISPR–CasΦ effector. Nat Struct Mol Biol 2021;28:652–61. https://doi.org/10.1038/s41594-021-00632-3.
